# Revealing the Hidden Electrochemical Pathway for Cathode Electrolyte Interface Formation in Lithium–Sulfur Batteries with Carbonate-Based Electrolytes

**DOI:** 10.1021/acsaem.5c02970

**Published:** 2025-12-15

**Authors:** Francisco J. García-Soriano, Jan Jerovsek, Santiago A. Maldonado-Ochoa, Fabian Vaca Chávez, Delvina Japhet Tarimo, Volker Presser, Bostjan Genorio, Marc Florent, Teresa J. Bandosz, Robert Dominko, Christian Prehal, Alen Vizintin

**Affiliations:** † 68913 National Institute of Chemistry, Hajdrihova 19, 1000 Ljubljana, Slovenia; ‡ 28217 Universidad Nacional de Córdoba, Facultad de Matemática, Astronomía, Física y Computación, Grupo de Resonancia Magnética Nuclear, Av. Medina Allende s/n, Ciudad Universitaria, X5000HUA Córdoba, Argentina; § Consejo Nacional de Investigaciones Científicas y Técnicas, CONICET, IFEG, Av. Medina Allende s/n, Ciudad Universitaria, X5000HUA Córdoba, Argentina; ∥ 28391 INM - Leibniz Institute for New Materials, Campus D22, 66123 Saarbrücken, Germany; ⊥ Department of Material Science and Engineering, Saarland University, Campus D22, 66123 Saarbrücken, Germany; # saarene - Saarland Center for Energy Materials and Sustainability, Campus C42, 66123 Saarbrücken, Germany; ∇ Faculty of Chemistry and Chemical Technology, 37663 University of Ljubljana, Večna pot 113, 1000 Ljubljana, Slovenia; ○ Department of Chemistry and Biochemistry, 14770 The City College of New York, 160 Convent Ave., New York, New York 10031, United States; ◆ Alistore-European Research Institute, CNRS FR 3104, Hub de l’Energie, Rue Baudelocque, 80039 Amiens, France; ¶ Department of Chemistry and Physics of Materials, 625366 University of Salzburg, Jakob-Haringer-Straße 2a, 5020 Salzburg, Austria

**Keywords:** lithium−sulfur batteries, cathode-electrolyte interphase, microporous carbon, carbonate-based electrolytes, polysulfides

## Abstract

This study investigates the role of microporous carbons and carbonate-based electrolytes in addressing challenges related to polysulfides dissolution and electrolyte compatibility in lithium–sulfur (Li–S) batteries. By employing microporous carbons and varying the sulfur content, we investigate the formation of the cathode-electrolyte interphase (CEI) during the first discharge process. We propose an electrochemical nucleophilic mechanism for the formation of the CEI involving polysulfides and solvent molecules in the confined small pores of the cathode. This interphase, primarily composed of LiF, effectively seals the carbon pores, preventing further solvent intrusion and stabilizing the system. Furthermore, it allows the use of wider pores without compromising the system. Our findings reveal that an increased sulfur content within the micropores enhances cycling stability, contradicting trends observed in ether-based systems. These insights highlight the potential of designing Li–S systems with optimized pore structures and electrolyte compositions to achieve greater stability and capacity retention, marking a significant step forward in the development of practical Li–S batteries.

## Graphical abstract

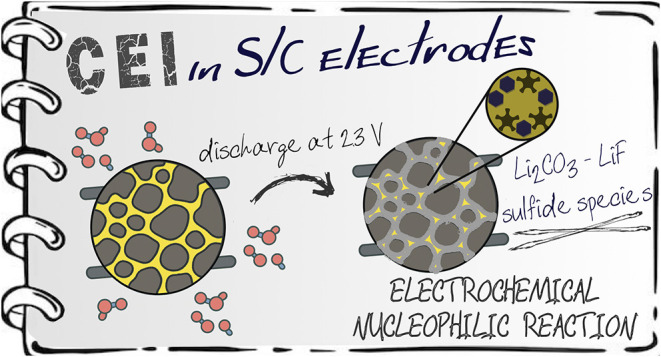

## 1 Introducion

Lithium–sulfur (Li–S) batteries are promising next-generation batteries due to their cost-effectiveness, environmental benefits, and high storage capacity. However, their widespread commercialization has been hindered by short cycle life and challenges in reaching their theoretical capacity. A key issue arises from the interaction between polysulfides and solvent molecules in carbonate-based electrolytes, leading to a nucleophilic attack that results in complex degradation pathways and capacity fade. This has largely confined research efforts to ether-based electrolytes.^1,2^ To address this challenge, recent strategies have focused on the use of (ultra)­microporous carbons with pore sizes similar to those of solvent molecules, which can be suitable for carbonate-based electrolytes.^3–6^ This design ensures that sulfur lithiation proceeds via a solid-state mechanism, as evidenced by a single plateau observed in charge/discharge profiles. However, it has been reported that the use of carbons with larger pores and employing carbonate-based electrolytes also led to long-term stability.^7–9^

The first electrochemical discharge in Li–S batteries using carbonate-based electrolytes typically exhibits two distinct plateaus. The high-voltage plateau, around 2.4 V vs Li/Li^+^, has been widely reported in the literature,^5,7,10,11^ although its presence is not universal.^4,8^ In ether-based electrolytes, this plateau is associated with polysulfide formation in solution.^12,13^ However, for carbonate-based systems, the mechanism remains debatable. Some works attribute this plateau to the formation of polysulfides outside the pore structure.^5,10^ In contrast, others suggest that interactions between the electrolyte and the carbon could play a critical role, possibly linked to the formation of a cathode-electrolyte interphase (CEI).^3^ Recent advances have provided deeper insights into this phenomenon.^14^ For instance, through *operando* neutron scattering and small-angle X-ray scattering, Gungor et al. provided unambiguous evidence that the CEI forms within the micropores of the carbon host, creating a nanoscale interphase that grows in the cathode’s internal pore structure.^15^ This discovery mandates a reinterpretation of the cathode’s electrochemistry: the CEI is not only on the external particle surface, but also inside the vast internal surface area of the pore network. These findings have raised fundamental questions about the nature, formation mechanisms, and functional role of the CEI in Li–S batteries, particularly in stabilizing carbonate-based electrolyte systems.

In this study, we investigate the CEI in microporous carbon–sulfur cathodes using a carbonate-based electrolyte. We propose that the CEI formation is driven by an electrochemical nucleophilic reaction between polysulfides and solvent molecules, occurring simultaneously with polysulfide generation during the first discharge plateau at 2.4 V vs Li/Li^+^. The critical feature for this is sulfur, located in the smaller pores of the carbon. These processes not only initiate the CEI formation but also contribute significantly to the charge consumption observed in this voltage region. Through an X-ray photoelectron spectroscopy (XPS) analysis, we elucidate the chemical composition of the CEI, revealing that it is predominantly composed of lithium fluoride (LiF), lithium carbonate (Li_2_CO_3_), and sulfide species. This LiF layer effectively seals the pores of the carbon host, enabling the utilization of carbons with pore sizes broader than the molecule solvents. Furthermore, these findings provide critical insights into the design of next-generation Li–S batteries, offering improved cycle life and stability in carbonate-based electrolytes.

## 2 Experimental Section

### 2.1 Materials and Synthesis

The microporous carbon (MC) powder from SAFT was combined with elemental sulfur (Sigma-Aldrich) with different sulfur mass loadings (20, 35, 50, and 65 wt % sulfur) and subjected to ball milling at 300 rpm for 30 min. The resulting mixtures were transferred to borosilicate glass vials for thermal treatment using a Büchi glass oven. This infiltration process, consistent with previous procedures,^16^ ensures that sulfur is confined within the carbon pore structure, avoiding the presence of external sulfur. The resulting carbon–sulfur (C/S) composites were labeled MC-S20, MC-S35, MC-S50, and MC-S65, corresponding to their respective sulfur contents. Pure MC and pure sulfur were also tested as control samples, labeled as MC-S0 and S100, respectively. All MC-S composites were used as active materials in Li–S batteries with carbonate-based electrolyte.

### 2.2 Materials Characterization

The sulfur content of the MC-S composites was determined using thermogravimetric analysis (TGA) on an STA 449 F3 Jupiter. The analysis was conducted under an argon atmosphere, with a heating rate of 10 °C min^–1^, up to a maximum temperature of 900 °C. Porosity characterization was performed using nitrogen gas sorption analysis (GSA) at −196 °C on an Autosorb iQ system (Quantachrome, now Anton-Paar). Before analysis, the MC sample was degassed at 200 °C for 12 h under vacuum while the MC-S samples were degassed at 100 °C for 24 h. The pore size distribution was determined using quenched solid density functional theory (QSDFT), assuming a slit-shaped pore geometry.^17–19^

Small-angle X-ray scattering (SAXS) was carried out on the Xeuss 3.0 HR laboratory SAXS system from Xenocs, using a two-dimensional (2D) areal SAXS detector (Eiger 2R 1M, Dectris) and a Cu Kα X-ray microsource. The 2D data were azimuthally averaged and normalized by transmission values. As the effective thickness of the carbon/sulfur powder is difficult to determine experimentally, the SAXS intensities were normalized to the particle scattering at low *q* (*q* < 0.1 nm^–1^), assuming that the particle scattering must scale with the square of the mean electron density of the C/S particle and hence increase with increasing sulfur content. Scanning electron microscope (SEM) images were performed in an FE-SEM, Supra 35 VP Carl Zeiss, and an energy dispersive spectrometer (EDS) with Ultim Max 100 (Oxford, UK).

Nuclear magnetic resonance (NMR) experiments were conducted to analyze the interactions between the MC-S composites and the electrolyte. The MC-S powders were mixed with the same electrolyte used in the electrochemical experiments. The electrolyte volume was set to 1.1 times the free pore volume of the MC-S samples, as determined by GSA, ensuring that all micropores can be filled with the electrolyte. Sample preparation was performed in an argon-filled glovebox to prevent exposure to air or moisture. The prepared samples were transferred into zirconia NMR rotors, maintaining the inert atmosphere. One-dimensional (1D) ^1^H and ^7^Li NMR spectra were recorded at room temperature using a Bruker AVANCE II spectrometer, operating at 300.1 MHz for ^1^H and 116.6 MHz for ^7^Li. The experiments were conducted using a 4 mm magic angle spinning (MAS) probe at a spinning rate of 10 kHz. The spectra were acquired using π/2 pulses of 5.4 μs for ^1^H and 3.6 μs for ^7^Li. Adamantane (1.9 ppm) and LiCl (−1.1 ppm) were used as external chemical shift references for ^1^H and ^7^Li, respectively.

X-ray photoelectron spectroscopy (XPS) was used to analyze the MC-S cathodes at different stages of the first discharge. Samples were collected at 2.3, 2.1, and 1.8 V vs Li/Li^+^ after galvanostatic cycling under the same conditions as the electrochemical tests. To prevent contamination, cells were disassembled in an argon-filled glovebox, and the cathodes were dried under dynamic vacuum overnight. The samples were then transferred to the vacuum transfer module of the Versaprobe 3 AD spectrometer, preventing them from any contact with air. The XPS measurements were performed using a Versaprobe 3 AD (Phi, Chanhassen, US) equipped with a monochromatic Al–K_α1_ X-ray source (1486.6 eV) operating at 50 W. All spectra were recorded at room temperature under ultrahigh vacuum (10^–9^ mbar). The beam size was set to 200 μm, and a 1 mm^2^ area was scanned for each sample. Since the samples were mounted on nonconductive double-sided tape, a charge neutralizer (1 V, 20 μA) was used to prevent charging effects and ensure accurate measurement of binding energies. The analysis included survey scans as well as core-level spectra for phosphorus (P 2*p*), sulfur (S 2*p*), carbon (C 1*s*), oxygen (O 1*s*), and fluorine (F 1*s*). Survey scans were collected with a pass energy of 224 eV and an energy resolution of 0.8 eV, while core-level spectra were recorded with a pass energy of 27 eV and a resolution of 0.05 eV. The spectral deconvolution was performed using Voigt functions and a Shirley background correction, both implemented via the Ulvac-PHI Multipak software. For energy calibration, a sputtered gold reference (Au 4*f* = 83.99 eV) was used to ensure accurate binding energy alignment.

### 2.3 Electrode Preparation

Cathodes were prepared by mixing the MC-S composites with polyvinylidene fluoride (PVDF) as a binder and carbon black (C65, Imerys) as a conductive agent in an 8:1:1 weight ratio. *N*-methyl-2-pyrrolidone (NMP) was used as the solvent. The resulting slurry was mixed in a planetary ball mill at 300 rpm for 30 min and then immediately cast onto carbon-coated aluminum foil (Armor, France). The coated foils were dried overnight at 80 °C, punched into circular electrodes (2 cm^2^), and then further dried at 50 °C for 48 h to remove any remaining adsorbed water. The sulfur loading was fixed at 1 mg cm^–2^ for all electrodes.

### 2.4 Cell Assembly

Pouch-type two-electrode cells were assembled in an argon-filled glovebox (MBraun, O_2_, and H_2_O < 0.1 ppm) using MC-S cathodes, lithium metal as a counter and reference electrode, and a Celgard 2320 separator. The electrolyte used was 1 M LiPF_6_ in a 4:1 volume ratio of dimethyl carbonate (DMC) and fluoroethylene carbonate (FEC). The electrolyte volume was in excess (>20 μL mg_AM_^–1^, where AM = active material = S or C when S = 0 wt %) in all cells to prevent electrolyte depletion. For cyclic voltammetry (CV), three-electrode pouch-type cells were assembled with a double-layer glass fiber separator (20 mm diameter) and a lithium metal reference electrode placed between the two separators. The reference electrode was connected via a current collector located at the perimeter of the working and counter electrodes (*Supporting Information*, Figure S1). All chemicals used for the electrode preparation and cell assembly were obtained from Alfa Aesar.

### 2.5 Electrochemical Measurements

Galvanostatic charge/discharge measurements were performed using a Maccor 4200 potentiostat/galvanostat (Maccor, Inc.). Cells were cycled at a *C*/20 rate (*C* = 1.672 A g^–1^) within a voltage window of 1 to 3 V vs Li/Li^+^. Cyclic voltammetry (CV) was carried out at scan rate of 0.1 mV s^–1^ using a Biologic VMP3 potentiostat. The electrochemical voltage data is referenced against the Li/Li^+^ redox couple. Therefore, all reported potentials are relative to Li/Li^+^, even if not explicitly stated.

Symmetrical electrochemical cells were assembled using two nearly identical preconditioned cathodes to isolate the impedance contribution of the cathode interface. Cathodes were prepared at two specific states of discharge (SoD): precycled and discharged to 2.1 V. These cathodes were first conditioned in standard full-cells with a lithium metal anode, including a 5-h rest period and, for the 2.1 V SoD, a galvanostatic discharge at *C*/20. The cells were then disassembled in an argon-filled glovebox, and the cathodes were retrieved to construct symmetrical cells using a fresh Celgard separator and an additional 10 μL mg_S_^–1^ of electrolyte. This configuration was chosen specifically to eliminate the nontrivial and variable impedance contribution of the lithium metal anode.^10,20^ Potentiostatic electrochemical impedance spectroscopy (EIS) was performed on the symmetrical cells at 0 V (vs the two identical electrodes) with a 10 mV (rms) perturbation amplitude over a frequency range of 10 kHz to 1 mHz using a Biologic VMP-3 potentiostat/galvanostat.

## 3 Results and Discussion

### 3.1 Materials Characterization

To determine their pore structure, the MC and MC-S materials were analyzed using N_2_ adsorption/desorption. The type II isotherms confirm that the MC is exclusively microporous (in *Supporting Information*, Note S1, Figure S2A). Specifically, MC-S0 exhibits two distinct types of micropores, with average widths of 0.85 and 1.68 nm, as determined by QSDFT pore size distribution calculations (Figure 1A, Figure S2B).

**Figure 1:**
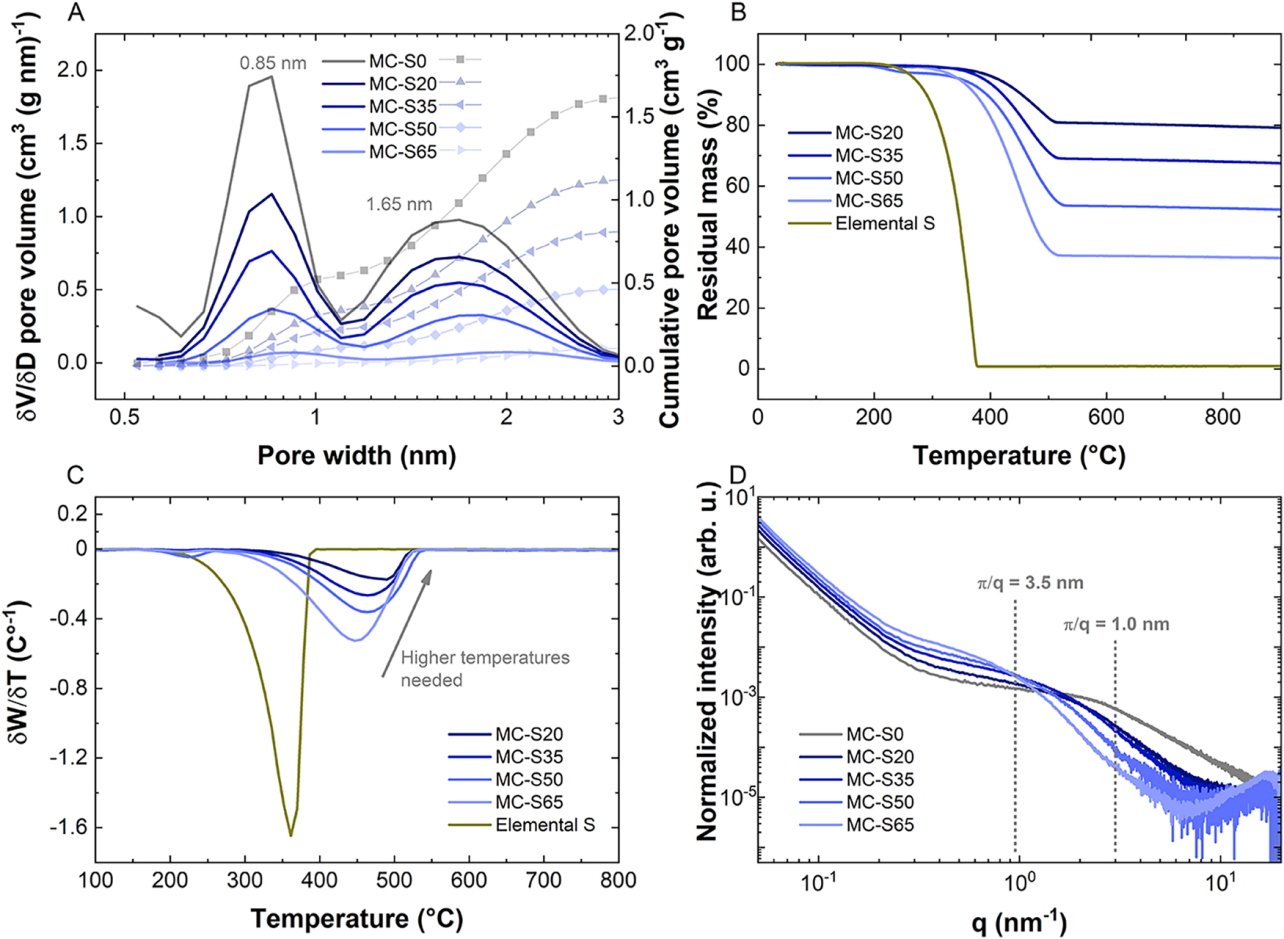
(A) Differential pore size distribution (left side) and cumulative pore size distribution (right side); the values correspond to the percentage of pore filling at each pore size, relative to the bare carbon, (B) thermogravimetric curves measured for sulfur and the MC-S powder, (C) differential thermogram, (D) small-angle X-ray scattering data.

The larger pores have approximately three times the size of FEC and DMC molecules.^21^ Based on the total pore volume of MC-S0 and the density of liquid sulfur (1.82 g cm^–3^), the theoretical maximum sulfur infiltration is estimated to be 73 wt %. However, preliminary experiments reveal that the material can only accommodate up to 63 wt % sulfur, which is likely due to partial pore blocking or structural limitations.

The sulfur content in the materials was quantified by TGA (Figure 1B), which clearly showed that sulfur is located exclusively within the micropores. This is evidenced by the absence of significant weight loss at the sulfur evaporation point (350 °C). Instead, sulfur removal occurs at higher temperatures, and notably, the removal temperature increases as the sulfur content decreases (Figure 1C). This is due to sulfur’s preference for occupying the smallest micropores, which is attributed to its hydrophobicity and strong adsorption potential, aligning with energy minimization principles.^15,22^ *Supporting Information*, Figure S3, illustrates how both types of micropores become progressively filled as the sulfur content increases, with a slight preference observed for pores with a width of 0.85 nm. Qualitatively, the SAXS data (Figure 1D) also indicate that smaller pores fill first. However, analysis of the micropore position reveals an intensity shoulder in the region from 3.0 to 0.9 nm^–1^, indicating structural features around 3.5 nm that are larger than the pores themselves. We speculate that this unusual pattern arises from the very mobile (or even liquid-like) behavior of sulfur in contact with carbon, potentially forming particles approximately 3.5 nm in size, encapsulating the existing carbon nanostructures.^23^ However, conclusive verification of this morphology would require further dedicated research. TGA measurements in Figure 1B,C confirm that the corresponding sulfur is inside the particles. A more detailed SAXS analysis corroborates the pore-filling process (Figure 1D). The SAXS intensity profile of empty carbon exhibits three distinct regions.^19,24^ In the low-q regime (*q* < 0.2 nm^–1^), particle scattering dominates. The intermediate q-regime (0.2 nm^–1^ < *q* < 10 nm^–1^) features a distinct intensity shoulder, corresponding to nanopore scattering. In the high-q regime (*q* > 10 nm^–1^), the intensity is governed by the scattering of the carbon atomic structure factor. The intensity shoulder in the intermediate *q*-regime bends downward at around 3 nm^–1^, indicating a mean pore size of approximately π/*q* ≈ 1 nm. While the intensity shoulder represents scattering from all nanopores, the contribution is size-dependent: at higher *q*-values, smaller pores dominate, whereas at lower *q*-values, larger pores contribute more significantly.

Upon filling the carbon with sulfur, the SAXS intensity at low q increases, reflecting the rise in the mean electron density within the carbon particles. In the intermediate q-regime, the intensity shoulder shifts to lower *q*-values as the S/C ratio increases. Specifically, the intensity rises at lower *q*-values (<1 nm^–1^) and decreases at higher *q*-values (>1 nm^–1^). Since the SAXS intensity is proportional to the square of the electron density contrast between the carbon matrix and the pore volume, pore-filling leads to a reduction in the intensity. Thus, the observed trend supports a pore-size-dependent sulfur infiltration mechanism, where smaller micropores fill before larger ones.

High-magnification SEM images across increasing sulfur loadings (*Supporting Information*, Figure S4A–E) reveal that the characteristic morphology of the microporous carbon framework is well preserved from MC–S0 to MC–S65. At low magnification (≈10 μm), the particle size distribution, packing, and fracture facets remain essentially unchanged, with no evidence of bright, faceted secondary domains that would indicate the formation of micron-scale sulfur crystallites. At intermediate magnification (1 μm), the fracture surfaces and interparticle necks appear continuous, without the emergence of bridge-like deposits or film-like layers. Even at the highest magnification (200 nm), the surface microtexture remains distinct, showing only a slight smoothing at the pore mouths as sulfur loading increases, consistent with progressive in-pore filling rather than external deposition.

In agreement with these observations, SEM–EDX elemental maps (Figure 2A–E) acquired at identical magnification confirm a uniform distribution of sulfur throughout the carbon particle ensemble for MC–S20, MC–S35, MC–S50, and MC–S65. The S K_α_ signal closely follows the spatial extent of the carbon backbone, exhibiting no pronounced “hot spots,” rims, or bead-like aggregates along particle boundaries or contact points. Oxygen is detected at low and spatially diffuse levels, characteristic of surface functional groups, and shows no correlation with sulfur distribution. The absence of localized sulfur enrichment, combined with the lack of crystalline sulfur features in SEM, provides strong evidence that sulfur is predominantly accommodated within the microporous carbon framework rather than deposited externally.

**Figure 2:**
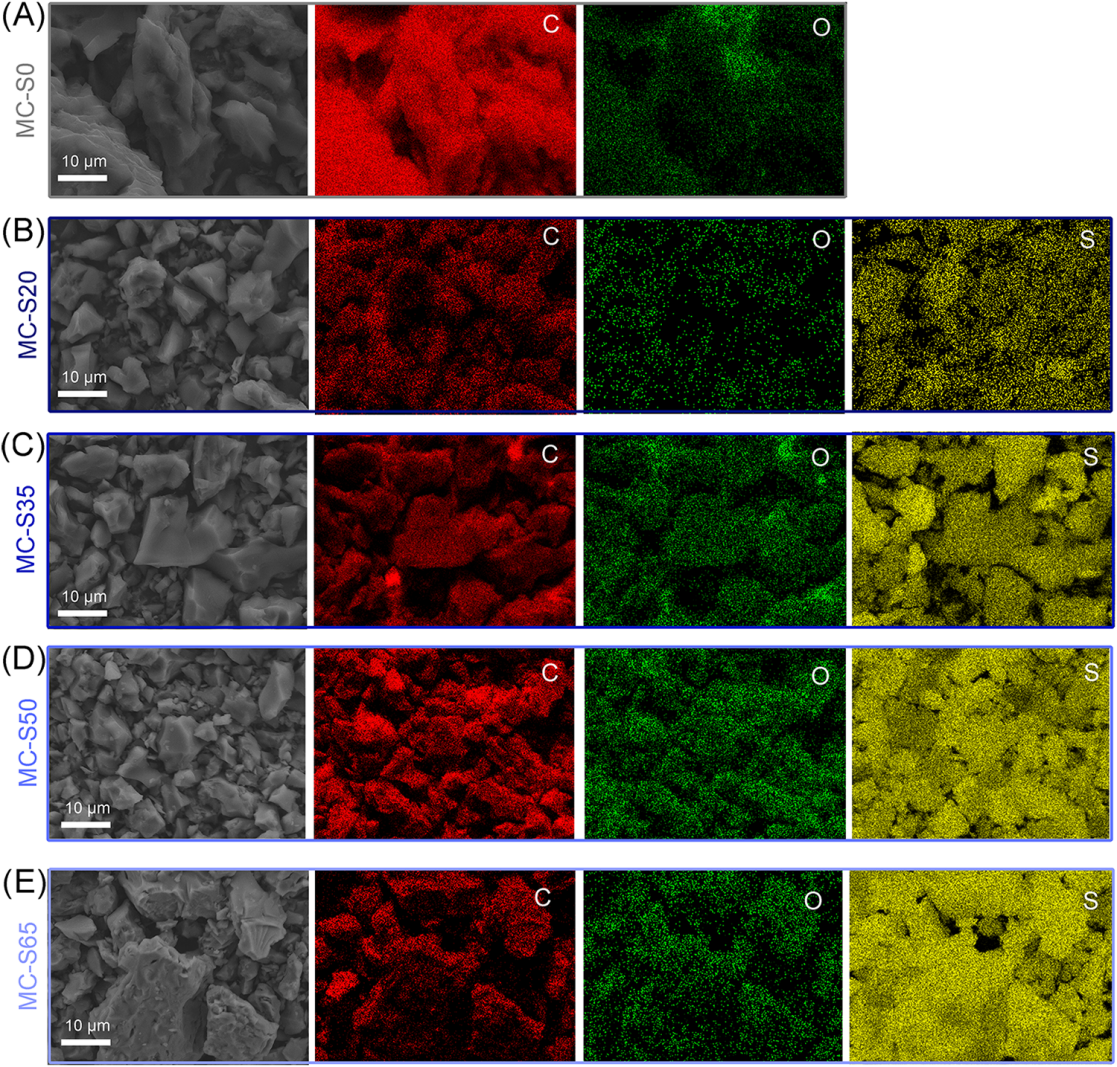
SEM–EDX elemental maps (carbon, oxygen, and sulfur) of (A) MC-S0, (B) MC-S20, (C) MC-S35, (D) MC-S50, and (E) MC-S65.

### 3.2 Electrochemical Characterization

Three-electrode cyclic voltammetry was carried out (Figure 3A) to identify distinct redox events. The cyclic voltammograms reveal three clear reduction peaks: (1) a peak at 2.4 V present in all samples; (2) a peak at 1.8 V, observed for MC-S20 and the MC-S0; and (3) a peak at 1.6 V, detected in MC-S65 and MC-S50 (although the latter is shifted to lower potentials). In literature,^4,5,7,8,10,11^ the reduction peak at 2.4 V has been assigned to polysulfide (PS) formation or interaction between the solvent molecules and the carbon structure. The peak at 1.85 V is associated with a reaction occurring on the bare carbon surface (MC-S0), which is more significant in MC-S20 due to its higher proportion of exposed carbon surface and may also be overlapping with the 2.4 V reduction peak. This peak is absent in samples with higher sulfur loading. The last reduction peak corresponds to the solid-state conversion of sulfur into Li_2_S.^6,15^ On the oxidation side, oxidation peaks are only observed for MC-S50 and MC-S65, located at 2.60 and 2.35 V, respectively. No oxidation peaks are detected for the other samples, this may be attributed to irreversible sulfur consumption during the first peak (higher area) and/or inaccessible sulfur trapped behind the CEI layer.

**Figure 3:**
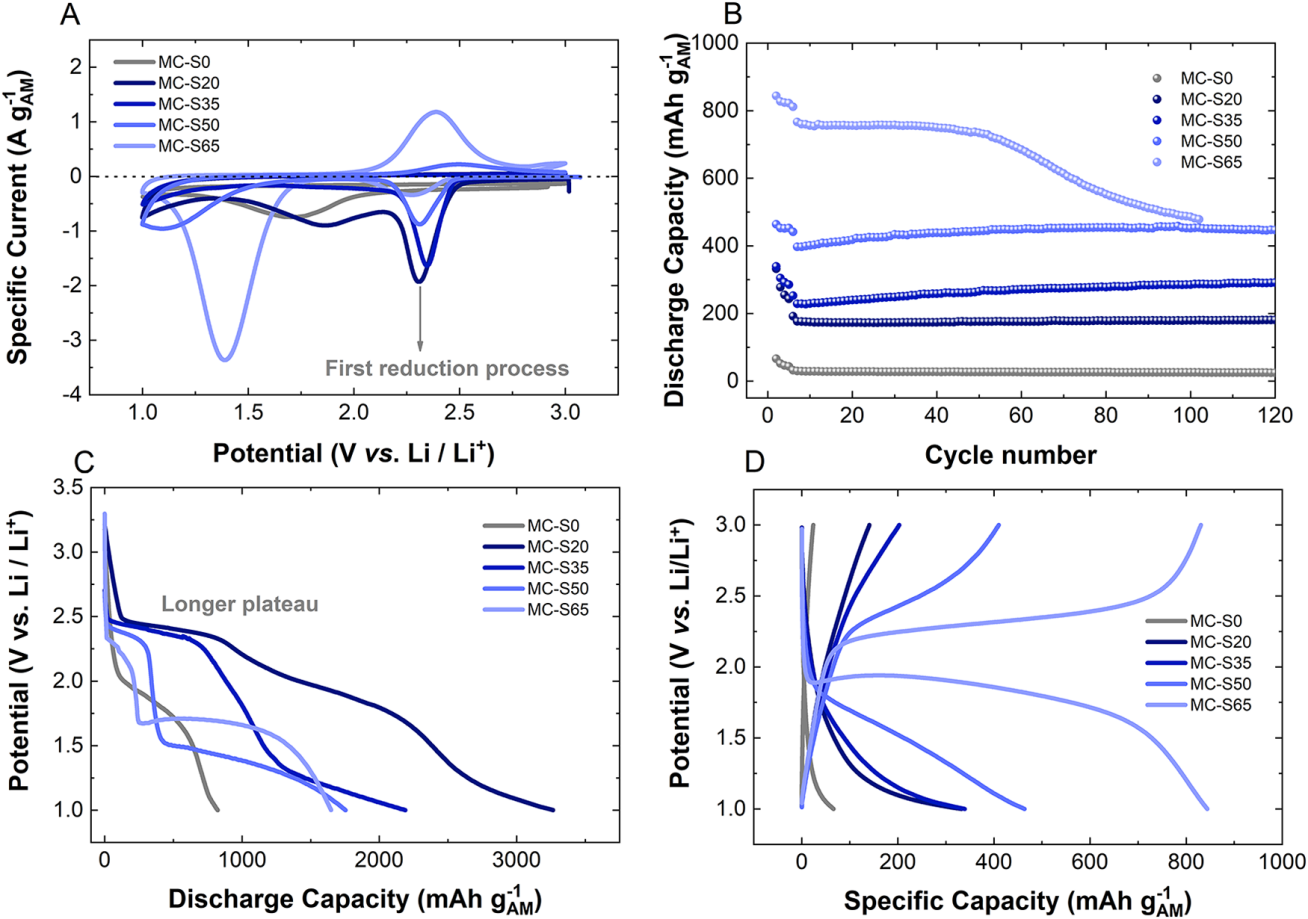
(A) Cyclic voltammograms of the first cycle at 0.1 mV s^–1^; (B) cycling stability at *C*/20 (cycles 2 to 5) and C/10 from cycle 6 onward; (C) first discharge profile at *C*/20; and (D) charge/discharge profiles at *C*/20 of the Li–S cells using MC-S cathode materials.

Galvanostatic charge/discharge experiments were conducted to verify these observations. The cycling stability is shown in Figure 3B, with the corresponding first-cycle discharge (Figure 3C) and charge–discharge profiles from the second cycle (Figure 3D). Additional distinctions between the samples can be observed. The first discharge profile shows a distinctive plateau around 2.4 V for all the samples with sulfur; the length of this plateau increases as the sulfur content decreases in the carbon host. During the initial charge, only MC-S50 and MC-S65 show a plateau around 2.35 V. In contrast, during the subsequent discharge, plateaus emerge at 1.75 V (UMC-S50) and 1.85 V (MC-S65) (Figure 3D). Interestingly, MC-S65 exhibits a lower overpotential and achieves a higher discharge capacity compared to the other MC-S cathodes. This may be related to the improved charge transfer efficiency at the carbon-active material interface.^15^ Notably, MC-S65 achieves approximately 800 mAh g^–1^ over 60 cycles, whereas MC-S50 provides around 450 mAh g^–1^, maintained beyond 120 cycles (Figure 3B). The capacity fading observed in the MC-S65 cathode is most likely attributed to FEC depletion and lithium anode passivation rather than a cathodic issue.^16^ This suggests that the degradation primarily occurs at the anode, likely due to the continuous formation of an unstable solid-electrolyte interphase (SEI) and lithium dendrite growth rather than due to sulfur loss or cathode degradation, *Supporting Information*, Note 2. In contrast, MC-S20 and MC-S35 exhibit pseudocapacitive behavior with no evident plateaus, similar to the bare MC-S0 electrodes. This finding suggests a complex interplay between sulfur loading, the MC structure, and the electrochemical processes.

The origin of the plateau at 2.4 V remains unclear. One hypothesis suggests that it results from PS formation from sulfur outside the MC carbon.^5,10^ This aligns with the idea that dissolved PS interacts spontaneously and irreversibly with the electrolyte, explaining the disappearance of this plateau in subsequent cycles.^1^ However, if the plateaus were related only to the formation of soluble Li_2_S_8_ polysulfides, the theoretical capacity limit for this process would be 209 mAh g^–1^, as it involves one-eighth of the total reduction of sulfur to Li_2_S.^25^ Besides, all MC-S cathodes exhibit capacities that exceed this limit, challenging this interpretation. Another explanation proposed in the literature is that the interaction of the electrolyte with the MC carbon could contribute to this plateau.^3^ However, this fails to explain why the plateau appears in the presence of sulfur but is absent in the bare MC carbon electrode.

These observations raise several important questions about the different processes involved in this plateau. In particular, what is the nature of the electrochemical processes taking place within this voltage window (>2.30 V)? Additionally, why does the capacity of this plateau increase as the sulfur content decreases? Another key question is why this plateau is absent in the bare MC cathode. Furthermore, why do MC-S20 and MC-S35 fail to exhibit the second plateau (1.75–1.85 V) observed in MC-S50 and MC-S65? Understanding these aspects is crucial for unraveling the underlying mechanisms governing these electrochemical behaviors.

To address these questions, we present several key pieces of evidence and interpretations. First, the observed behavior is not related to polysulfide formation from residual sulfur outside the MC structure. The TGA confirms that sulfur is present within the micropores, and the presence of traces of sulfur outside the micropores cannot account for the obtained capacities. Furthermore, experiments using microporous carbons with a significant amount of residual sulfur outside the carbon structure follow the same trend, as explained in *Supporting Information*, Note S3.^22^ Second, the processes are electrochemical. The large capacity contribution observed at 2.4 V requires a significant number of electrons, indicating that other electrochemical reactions, besides conventional polysulfide formation, are occurring. Third, although the processes achieve higher capacities at the lower sulfur content, a certain amount of sulfur remains essential as it is absent in the bare MC carbon electrode. This indicates that sulfur plays a critical role in activating this electrochemical process, possibly acting as a reaction intermediate or catalyst. Experiments with only 5 wt % of sulfur corroborate this hypothesis, achieving charge consumption higher than 2 Ah g^–1^ before the plateau at 1.8 V (*Supporting Information*, Figure S8). Moreover, the higher sulfur content improves the overall cathode performance. As the sulfur content increases, the discharge capacity increases, and the Li–S battery exhibits more defined plateaus. Finally, the symmetrical impedance spectra of the MC–S65 cathode (*Supporting Information*, Figure S9) show a clear decrease in the high-frequency semicircle after the first plateau. This feature implies a substantial enhancement in interfacial charge transport, which can be attributed to the formation of an ionically conductive cathode–electrolyte interphase (CEI) within the carbon pores. Rather than hindering charge transfer, this interphase appears to establish a functional interface that facilitates Li^+^ transport and electron exchange. These findings collectively point to specific mechanisms for CEI formation during the first plateau.

### 3.3 Electrochemical Nucleophilic Reaction with Carbonate Electrolytes

Considering the above discussion, we propose an electrochemical nucleophilic reaction between polysulfides and the solvent molecules of the electrolyte inside the micropores of the MC. The nucleophilic attack is the mechanism by which PS spontaneously decomposes carbonate electrolytes, and it is the main reason why carbonate-based electrolytes are unsuitable for traditional Li–S batteries. However, unlike the purely chemical decomposition of solvents, here we propose an electrochemical route where electron transfer occurs and contributes to the charge consumption of the first discharge plateau. This process is facilitated by the presence of polysulfides, which act as redox mediators. Two reduction pathways are proposed, one for FEC and the other for DMC, as can be seen in the following equations

1 $$FEC+e^{-}+{Li}^{+}+PS\to LiF+{CO}_{2}+R\text{−}S\text{−}R+{CH}_{2}\text{−}CHF$$

2 $$DMC+e^{-}+{Li}^{+}+PS\to {Li}_{2}{CO}_{3}+{CH}_{4}+DMS$$

In the FEC reduction pathway, FEC reacts with an electron (e^–^), a lithium ion (Li^+^), and polysulfides to produce primary products: lithium fluoride (LiF), carbon dioxide (CO_2_), thioesters (R–S–R), and organic fluorides, such as CH_2_–CHF. Similarly, in the DMC reduction pathway, DMC reacts with an electron, a lithium ion, and polysulfides to yield three key products: lithium carbonate (Li_2_CO_3_), methane (CH_4_), and dimethyl sulfide (DMS), which is a thioester (R–S–R). These electrochemical reactions occur inside the pore structure, where polysulfides (S_*x*_^2–^) act as catalytic mediators, enabling charge transfer. The stable capacity retention over subsequent cycles (Figure 3B) provides direct electrochemical evidence of effective pore sealing by the CEI, which prevents the dissolution and loss of active material.^26–28^ The presence of PS confined in small pores enables the reduction of carbonate solvents at lower potentials than would otherwise be required, as the PS species could reduce the energy barrier for the reaction. Moreover, ultramicropores may serve as nanoreactors or pseudocatalysts, facilitating chemical transformations and shifting reaction equilibria due to pronounced fluid–fluid interactions and confinement effects. The formation of solid CEI components (LiF and Li_2_CO_3_) and the evolution of gaseous or soluble byproducts (CO_2_, CH_4_, DMS) have significant implications for battery stability and performance. The CEI layer can protect the electrode and improve its stability; however, excessive CEI growth can block active sites on the electrode, thereby affecting its long-term performance. This mechanism underscores the importance of controlling the extent of carbonate decomposition in Li–S batteries.

The questions raised above can now be addressed based on the proposed mechanism. The electrochemical process occurring in this voltage window involves the generation of polysulfides, which actively participate alongside solvent molecules in an electrochemical nucleophilic reaction. This reaction plays a crucial role in the formation of the CEI on these cathodes, influencing their electrochemical behavior and stability. The increase plateau capacity with decreasing sulfur content is due to the coexistence of the electrolyte and sulfur in smaller pores, with a greater volume of these pores available for the latter. Under these conditions, the interactions between solvent molecules (FEC and DMC) and the polysulfides are enhanced, promoting the nucleophilic electrochemical reaction. Since this reaction is pore size-/surface-mediated, the greater the extent of the carbon-electrolyte interface area is, the greater the resulting increase in plateau capacity becomes, as the S/C ratio decreases. A key aspect of this process is the necessity of confining sulfur in small pores for the reaction to occur. The presence of polysulfides (S_*x*_^2–^) is essential, as they act as mediators in the electron transfer process. Without sulfur, no polysulfides are formed, and thus, the reaction does not occur. This explains the absence of this behavior in bare MC cathodes (MC-S0). The polysulfides effectively lower the activation energy required for the reduction of carbonate solvents, allowing the reaction to proceed at voltages as high as 2.4 V. The greater performance of MC-S65 compared to other MC-S is attributed to its higher sulfur content. In MC-S65, a significant fraction of the internal MC pore structure is filled with sulfur, and only the outer surface remains exposed to the electrolyte. This limits the electrochemical nucleophilic reaction to the outermost regions of the MC particles. Further discussion about the reactions kinetics and the mechanism are in *Supporting Information*, Note 4.

In contrast, in MC-S50, MC-S35, and MC-S20, the electrolyte is exposed to small pores and their higher volume, which facilitates the formation of a thicker CEI throughout the interior of the carbon, leading to more severe pore clogging. This is so extreme in the MC-S35 and MC-S20 cathodes that the cells, after the first discharge, exhibit pseudocapacitive behavior. Instead, for the MC-S65, CEI formation is limited to the outermost surface, preventing an excessive blockage of the inner pores and allowing sulfur to remain accessible and CEI-protected for subsequent cycles.

To support the proposed reaction mechanism, it is crucial to demonstrate that solvent molecules penetrate the micropores of the carbon structure, where they can interact with polysulfides. This can be achieved using solid-state nuclear magnetic resonance (ssNMR) spectroscopy, a technique well-suited to detect the presence of confined molecules in porous environments.^29–32^ It is well-established that the NMR spectra of species confined within microporous carbons shift to lower chemical shift (δ) values compared to their bulk counterparts.^32^ For a set of samples with the same graphitization degree and identical confined molecules, the spectra are mainly determined by two factors: (1) the pore size distribution, where the larger shifts correspond to molecules confined in smaller pores, and (2) the mobility of the molecules, with rapid exchange between sites averaging the signals.^32,33^

Figure 4 compares the ^1^H and ^7^Li ssNMR spectra of the pure electrolyte (black) with those of the electrolyte/MC-S powder samples. Both for the ^1^H signal from the solvent molecules (Figure 4A) and for the ^7^Li signal from the cations (Figure 4B), the peaks of MC and MC-S samples are shifted to lower δ compared to the bulk spectra (black). This indicates that the electrolyte is confined in the pores. In addition, the spectra lack signals corresponding to ex-pore (outside the pores) at the bulk δ value. This observation suggests that the in-pore and ex-pore signals are averaged due to fast exchange.^30,33^

**Figure 4:**
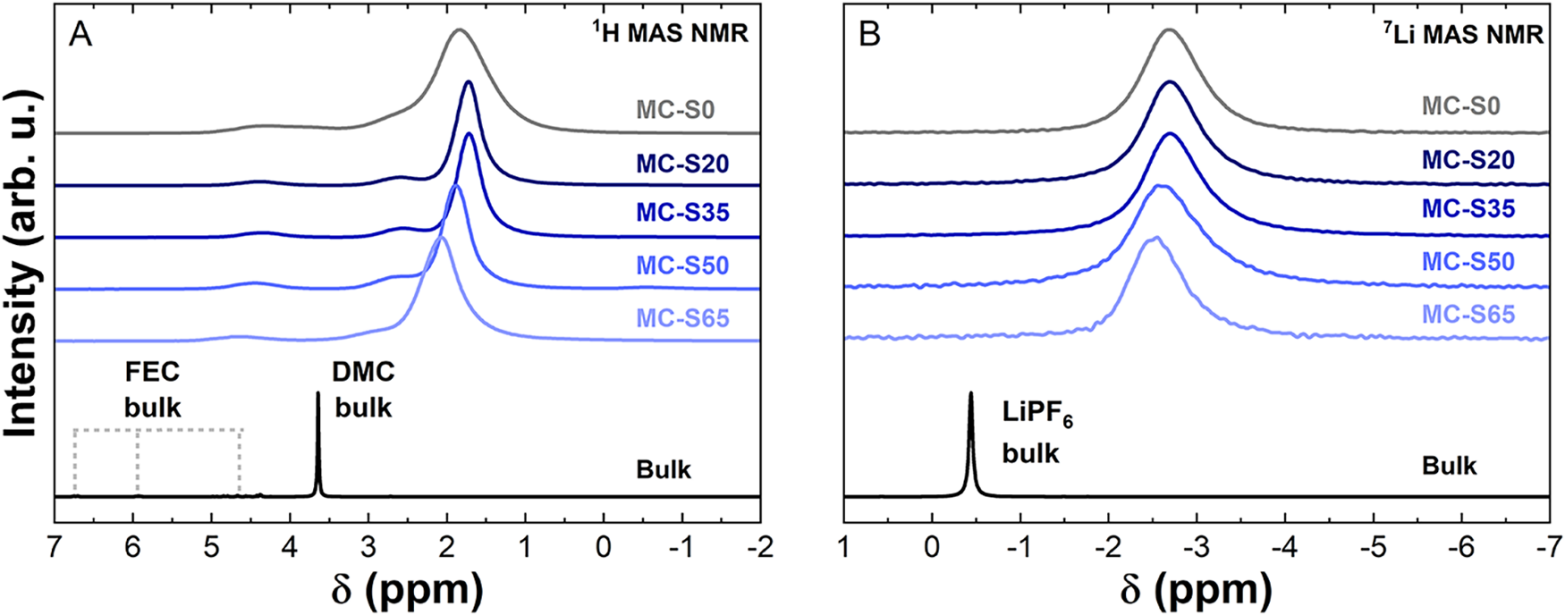
(A) ^1^H and (B) ^7^Li solid-state nuclear magnetic resonance spectra.

For ^1^H ssNMR, assigning confined peaks is challenging due to the multiple peaks in the bulk spectrum, particularly the FEC contribution (δ > 4 ppm), as well as the broadening upon confinement. However, the spectra can be analyzed by focusing on the DMC signal, characterized by the peak at δ ∼ 3.6 ppm in the bulk spectrum and the dominant signal at δ < 2 ppm in MC-S samples. This broad signal indicates a distribution of confinement environments consistent with solvent molecules occupying pores of various sizes. The major peak for the MC-S samples shifts to higher δ values as the sulfur content increases, moving from more confined (lower ppm) to less confined (higher ppm) environments. These observations align with the pore size distribution shown in Figure 1A, as the micropore volume ratio of 0.85 to 1.65 nm decreases with increasing sulfur loading (in *Supporting Information*, Note S1). This consistency validates the conclusion that the solvent molecules explore the free pore volume of the samples. Finally, the ^7^Li spectra of the electrolyte in MC-S exhibit the same trend as the ^1^H dominant peak, strongly suggesting that both the solvent and the cations are present in the micropores. These findings suggest that solvent molecules and cations can directly interact with PS within the micropores, facilitating the proposed electrochemical nucleophilic attack mechanism.

To further investigate the formation of the proposed species, XPS measurements were performed on the cathode surface after being discharged at three specific voltages: 2.35, 2.10, and 1.80 V. It is critical to note that for highly microporous carbons, where the pore walls constitute the vast majority of the surface area, the XPS signal (with a probing depth of around 10 nm) originates predominantly from these internal pores, not just the external particle surface. Given that our carbon features pores <2.5 nm, the analyzed volume is representative of the in-pore environment where the relevant chemistry occurs.^34,35^ The MC-S65 and MC-S20 cathodes were selected for this analysis to (a) understand the surface chemistry, (b) corroborate if the same species are formed in the different S/C ratios, and (c) track the formation of specific species at different stages of the discharge process. The XPS spectra of the MC-S65 cathode discharged to 2.35 V provide clear evidence of the presence of multiple chemical species (Figure 5). Detailed information on peak positions, full width at half-maximum (fwhm), and chemical assignments is available in *Supporting Information*, Note S5. The C 1*s* high-resolution spectrum reveals distinct signals, including a C–S bond at 285.9 eV, attributed to the decomposition of solvents that form R–S–R species such as DMS.^36–38^ This is further supported by the S 2*p* spectrum, where a corresponding contribution appears at 165.0 eV. The CH_2_ signal at 286.4 eV in the C 1*s* spectrum likely originates from CH_2_–CHF, a product of FEC decomposition, though it may also partially arise from PVDF.^39,40^ These two contributions can be distinguished in the F 1*s* spectrum: the signal for PVDF appears at 686.4 eV, while the CH_2_–CHF species is observed at 688.0 eV.^39,40^

**Figure 5:**
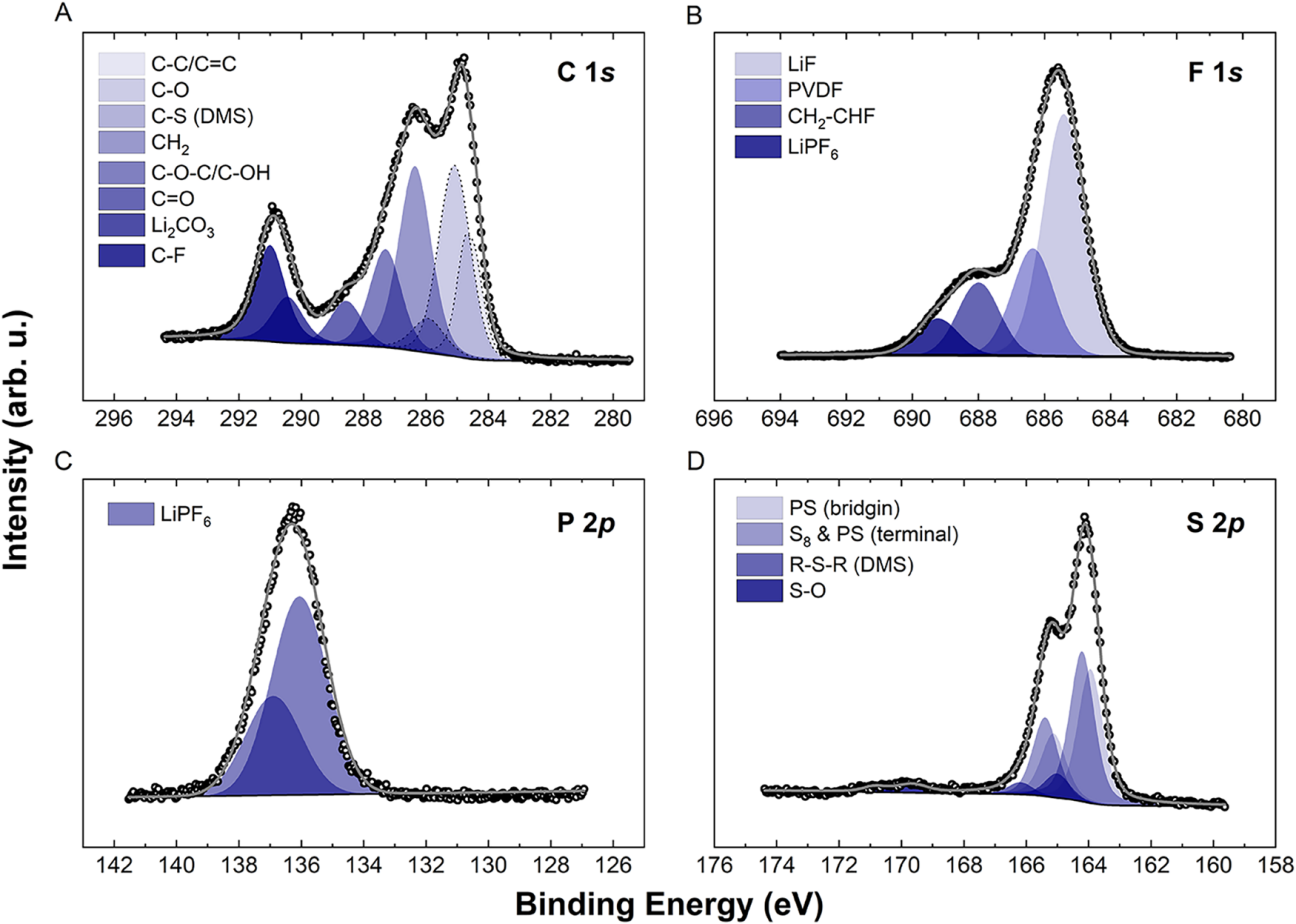
High-resolution X-ray photoelectron spectra and their deconvolution of the MC-S65 cathode after being discharged to 2.35 V; (A) C 1s, (B) F 1s, (C) P 2p and, (D) S 2p, where the black circles represent the acquired spectra, and the gray line shows the fit.

Another important contribution in the C 1*s* spectrum is at 290.5 eV, corresponding to lithium carbonate, formed through the DMC decomposition pathway.^37^ Most significantly, the F 1*s* spectrum confirms the formation of LiF at 685.4 eV, indicating FEC ring-opening reactions. This signal is notably more intense than that of the PF_6_^–^ anion at 689.3 eV, suggesting the formation of a substantial amount of LiF. Importantly, no evidence of salt decomposition is detected at this stage. This observation is corroborated by the P 2*p* spectrum, which features a single peak at 136.9 eV, attributed to the intact PF_6_^–^ anion, confirming that LiF formation results exclusively from the decomposition of FEC.^41^ In the S 2*p* spectrum, in addition to the C–S–C bond at 165.0 eV, polysulfides at 163.2 eV can also be identified. At 2.1 V, the spectra remain largely unchanged, with no additional species detected, suggesting that no new reactions occur within this voltage range. However, at 1.8 V, new contributions emerge in the spectra, particularly from salt decomposition products and Li_2_S (*Supporting Information*, Figure S11–S12). Two new signals from LiPF_6_ degradation can be distinguished, at 686.9 eV in the F 1*s* spectra and 135.5 eV in the P 2*p* spectra, corresponding to POF compounds.^41^ This highlights that salt decomposition processes are confined to lower voltages and do not influence the formation of species observed at higher potentials. These findings offer insight into the electrochemical mechanisms, showing that salt decomposition does not contribute to species formation during the initial stages of the discharge process.

The analysis of the MC-S20 samples confirmed the same findings observed for the MC-S65 samples (*Supporting Information*, Note 5). The spectra clearly show the presence of LiF, CH_2_–CHF, Li_2_CO_3_, and C–S–C bonds (likely from dimethyl sulfide). No additional chemical species were detected, indicating that the same electrochemical reactions occur in both cathodes. These results suggest that the amount of sulfur in the cathode (S/C ratio) does not alter the chemistry of the electrochemical solvent decomposition pathways, confirming that the same reactions take place across all systems studied. Therefore, the increase in the plateaus is due to a greater extent of the available volume in very small pores.

Overall, these results strongly support the proposed mechanism of an electrochemical nucleophilic reaction facilitated by polysulfides and solvent molecules within the micropores of the carbon host. The ssNMR evidence of solvent confinement, combined with the XPS detection of key chemical species such as LiF, R–S–R, and Li_2_CO_3_, provides a comprehensive picture of the CEI formation process. Furthermore, the absence of salt decomposition products at voltages above 1.8 V confirms that the observed reactions are not linked to salt degradation but rather to the interaction between PS and the solvent molecules. This deeper understanding of the CEI formation mechanism highlights the critical role of micropores, particularly in terms of their sizes and volumes, polysulfides, and solvent confinement, in controlling the electrochemical behavior of Li–S batteries with carbonate-based electrolytes. This reaction potentially seals the pores with LiF, effectively confining the sulfur molecules and enabling the use of microporous carbons with a broader pore size distribution. Finally, the insights gained into the CEI formation mechanism open new avenues for interface engineering. While this study demonstrates that pore structure and sulfur content are critical parameters for controlling the CEI, future work could explore the influence of the sulfur isotope itself (e.g., ^34^S) as they can significantly alter polysulfide solvation and migration kinetics.^42–44^

## 4 Conclusions

The findings of this study highlight the critical role of micropore size and confined sulfur content in small pores on the electrochemical performance of Li–S batteries using carbonate-based electrolytes. We found that the CEI formation is contingent on micropores larger than the solvent molecules, which explains why some (ultra)­microporous carbons do not exhibit this phenomenon. Increasing the sulfur content within the micropore structure enhances cycling performance, contrary to trends observed in ether-based systems. During the first discharge, the accessibility of pore volume to the electrolyte facilitates CEI formation and blocks further solvent intrusion. The CEI, primarily composed of LiF, Li_2_CO_3_, and sulfide species, effectively seals the pores, stabilizes the interphase, and allows the utilization of microporous carbons with pores larger than the previously stipulated 0.7 nm pores. Thus, a controlled deposition of sulfur in small pores “self-cures” the instability problems encountered in microporous carbon electrodes. In summary, our findings offer a fresh perspective on the design principles for advanced Li–S batteries utilizing microporous carbons. Specifically, (1) the initial stages of the first discharge at higher cell voltages determine the formation of the CEI/active material nanostructure within the micropores, thereby influencing the overall performance. (2) Minimizing the contact area between the electrolyte and sulfur enhances active material utilization and reduces overpotentials during subsequent cycling.

## Supplementary Material



## Notes

Nitrogen sorption data and pore-size analysis; SEM images; additional electrochemical testing (rate capability, cycling, STACs); Nyquist spectra; kinetic analysis; XPS methods, peak-fit parameters, and full high-resolution spectra; supplementary discharge profiles; tables of porosity and XPS deconvolution parameters (PDF)
