# Supplementary material for: Revealing the Hidden Electrochemical Pathway for Cathode Electrolyte Interface Formation in Lithium–Sulfur Batteries with Carbonate-Based Electrolytes
Source: ACS Appl Energy Mater. 2025 Dec 15;9(1):211–21. doi: 10.1021/acsaem.5c02970 (PMC12801194; doi:10.1021/acsaem.5c02970)
Supplement: Supplementary file 1 [file ae5c02970_si_001.pdf]

SUPPORTING INFORMATION

Revealing the Hidden Electrochemical Pathway for Cathode Electrolyte Interface Formation in Lithium-Sulfur Batteries with Carbonate-based Electrolytes

Francisco J García-Soriano[1,*], Jan Jerovsek[1], Santiago A. Maldonado-Ochoa[2,3], Fabian Vaca Chávez[2,3], Delvina Japhet Tarimo[4], Volker Presser[4, 5, 6], Bostjan Genorio[7], Marc Florent[8], Teresa J. Bandosz[8], Robert Dominko[1,9], Christian Prehal[10], Alen Vizintin[1,*]

1. National Institute of Chemistry, Hajdrihova 19, 1000, Ljubljana, Slovenia
2. Universidad Nacional de Córdoba. Facultad de Matemática, Astronomía, Física y Computación. Grupo de Resonancia Magnética Nuclear. Av. Medina Allende s/n, Ciudad Universitaria, X5000HUA, Córdoba, Argentina
3. Consejo Nacional de Investigaciones Científicas y Técnicas, CONICET, IFEG. Av. Medina Allende s/n, Ciudad Universitaria, X5000HUA, Córdoba, Argentina
4. INM - Leibniz Institute for New Materials, Campus D22, 66123 Saarbrücken, Germany
5. Department of Material Science and Engineering, Saarland University, Campus D22, 66123 Saarbrücken, Germany
6. saarene - Saarland Center for Energy Materials and Sustainability, Campus C42, 66123 Saarbrücken, Germany
7. Faculty of Chemistry and Chemical Technology, University of Ljubljana, Večna pot 113, 1000, Ljubljana, Slovenia
8. Department of Chemistry and Biochemistry, the City College of New York, 160 Convent Ave., New York, NY 10031, United States of America
9. Alistore-European Research Institute, CNRS FR 3104, Hub de l'Energie, Rue Baudelocque, 80039, Amiens, France
10. Department of Chemistry and Physics of Materials, University of Salzburg, Jakob-Haringer-Straße 2a, 5020 Salzburg, Austria

* Corresponding Authors:
Francisco J. García-Soriano (francisco.soriano@ki.si) and Alen Vizintin (alen.vizintin@ki.si)

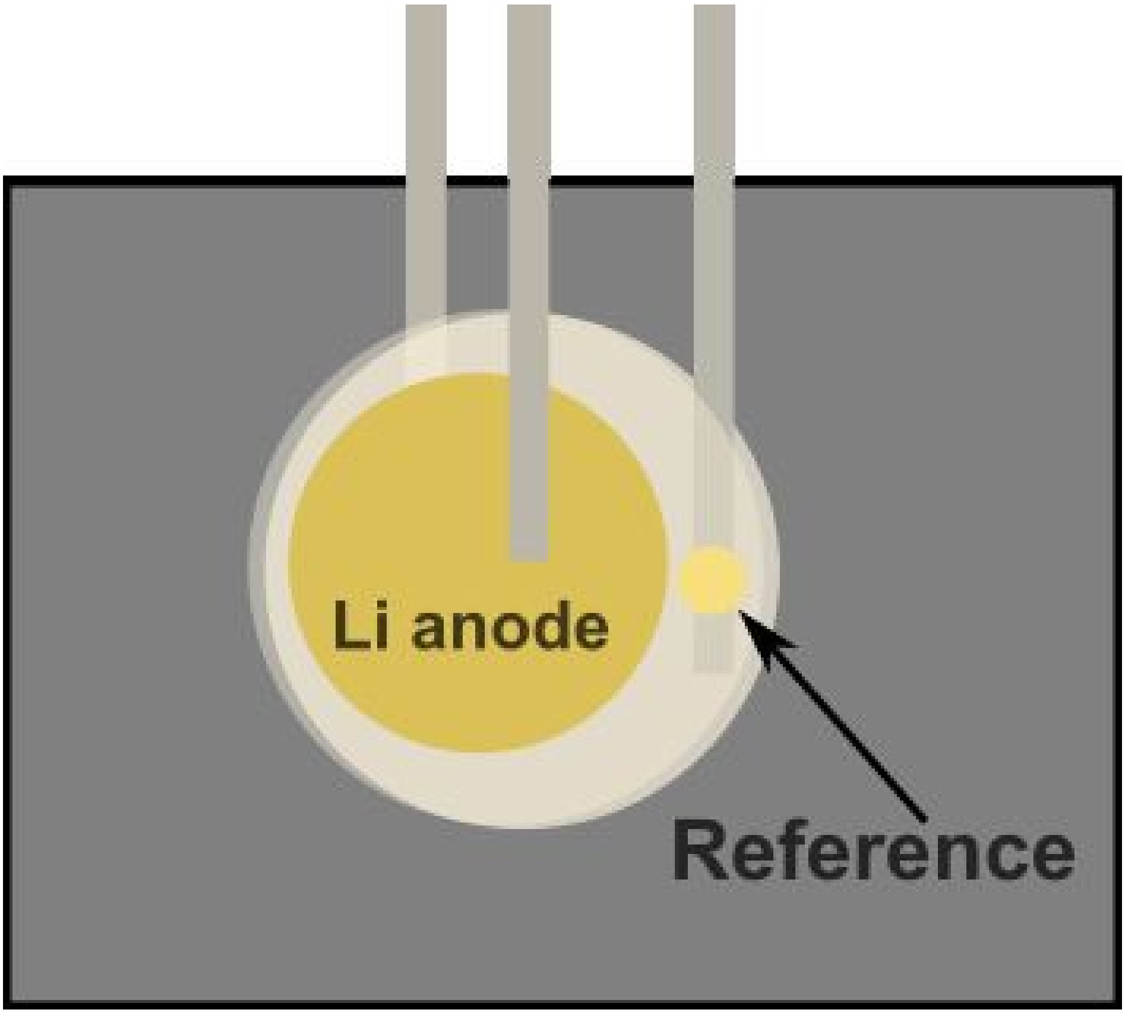

Figure S1. Schematics of the three-electrode cell used for electrochemical measurements.

Note 1: Nitrogen gas sorption analysis

Nitrogen gas sorption analysis was performed to determine the porosity characteristics of the investigated samples (MC-S0, MC-S20, MC-S35, MC-S50, and MC-S65). The N_2 isoterms are presented on **Figure S2A**. The cumulative pore volume data (**Figure S2B**) indicate significant differences among the samples. MC-S0 exhibited the highest cumulative pore volume (~1.8 cm³ g⁻¹), signifying a highly porous structure. Upon sulfur infiltration, a noticeable decrease in pore volume was observed with increasing sulfur content. MC-S20 and MC-S35 showed intermediate pore volumes (~1.2 and ~0.8 cm³ g⁻¹, respectively), whereas MC-S50 and MC-S65 presented the lowest pore volumes (~0.5 and ~0.3 cm³ g⁻¹, respectively). This reduction in cumulative pore volume indicates successful filling of the pores with sulfur, thereby confirming effective infiltration. The corresponding data table (**Table S1**) confirms the observed trend, clearly showing the decreasing pore volume as the sulfur content increases. These findings highlight the critical role of sulfur loading in tailoring the porosity and storage capabilities of the materials for practical applications.

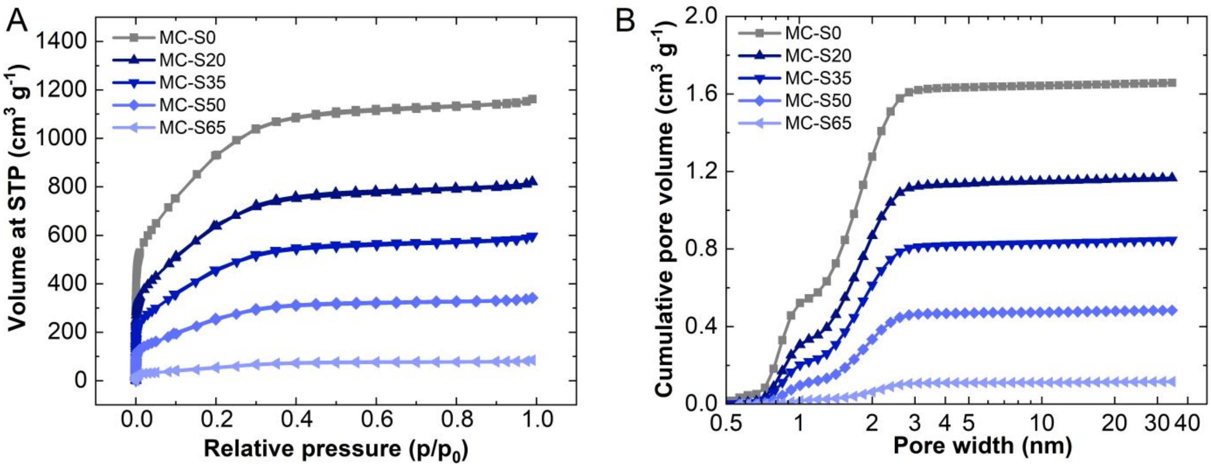

Figure S2. (A) N_2 physisorption isotherms of microporous carbons (and infiltrated samples) at 77 K and (B) pore volume with respect to pore width

Table S1. GSA analysis parameters

Name	DFT SSA ($m^2\ g^{-1}$)	Average pore size-d50 (nm)	Micropore volume ($cm^3\ g^{-1}$)	Mesopore volume ($cm^3\ g^{-1}$)	TPV ($cm^3\ g^{-1}$)
MC-S0	2511	1.4	1.28	0.38	1.66
MC-S20	1652	1.5	0.87	0.30	1.17
MC-S35	1161	1.5	0.62	0.23	0.85
MC-S50	627	1.7	0.33	0.15	0.48
MC-S65	135	1.8	0.06	0.05	0.12

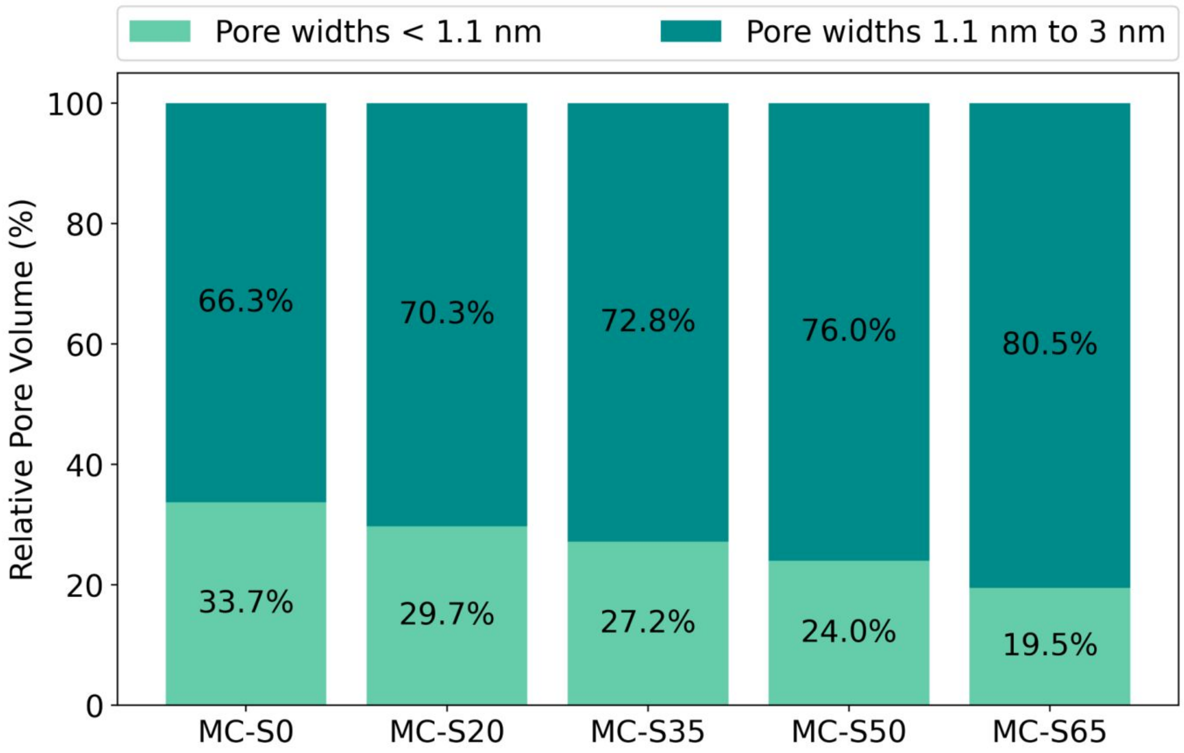

Figure S3. Relative pore volume obtained by integrating the contributions of each pore width from the bimodal pore size distributions shown in **Figure 1A.**

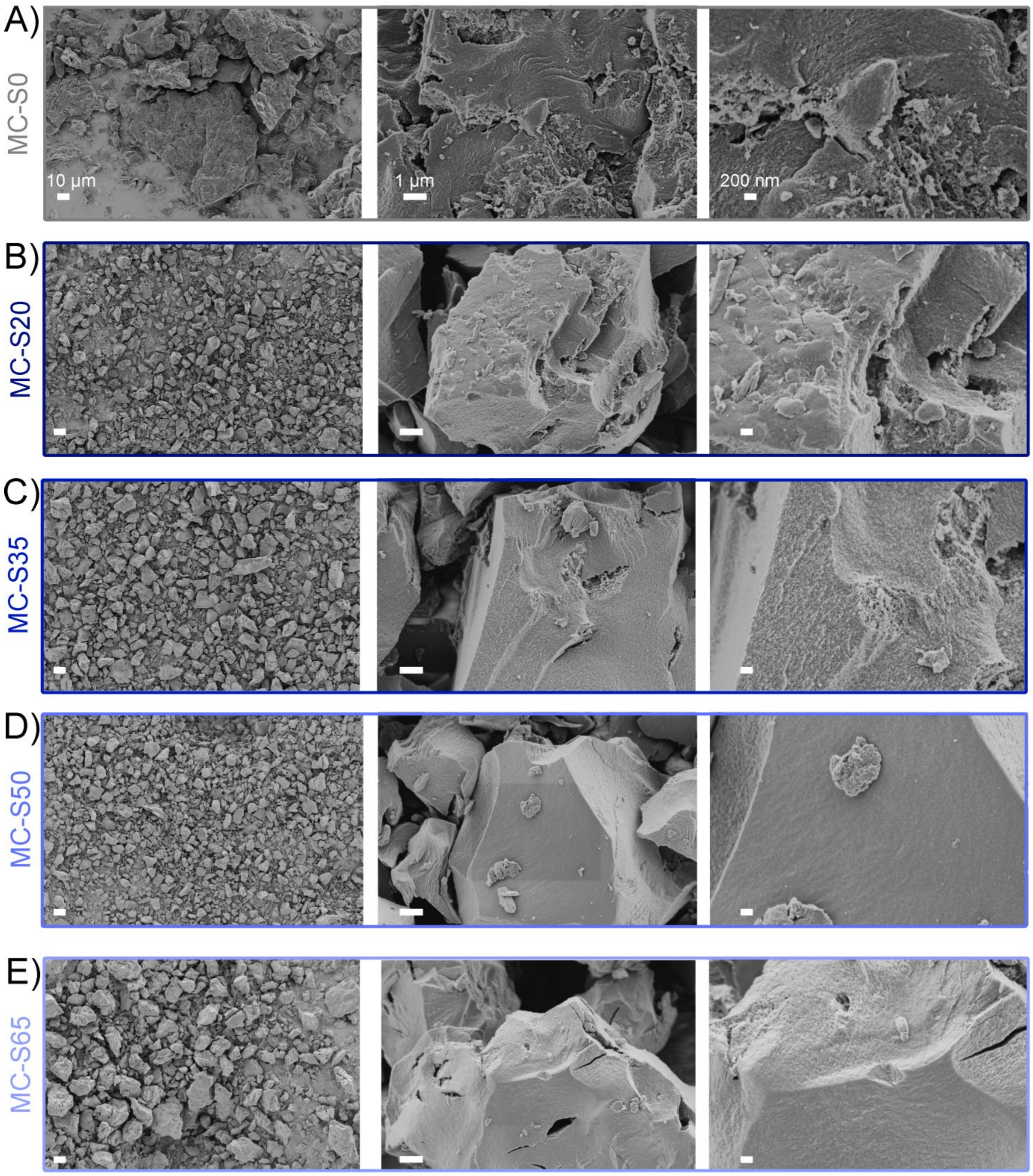

Figure S4: SEM of (A) pristine carbon (MC-S0) and carbon–sulfur composites ((B)MC-S20, (C) MC-S35, (D) MC-S50, and (E) MC-S65) at three magnifications (left to right: ~10 µm, 1 µm, 200 nm).

Note 2: Further electrochemical testing of the MC-S65 cathode: cycling stability and C-rate performance

Figure S5A displays the specific capacity of the MC–S65 cathode at various C-rates measured in a carbonate electrolyte. The electrode delivered an initial capacity of approximately 900 mAh g^{-1} at C/20. When the rate was increased, the specific capacity gradually decreased to about 800 mAh g^{-1} at C/10, 500 mAh g^{-1} at C/2, 450 mAh g^{-1} at 1C, and less than 100 mAh g^{-1} at 5C, indicating strong rate dependence likely due to mass transport limitations in the micropores of the C-S composite. Upon returning to C/10, the capacity is fully recovered, confirming the structural stability of the electrode and indicating that the cell does not exhibit a memory effect.
As established in the main text, the predominant failure mechanisms in these cells are related to lithium metal anode degradation and electrolyte depletion. To further substantiate this point, additional cycling experiments were performed using a higher electrolyte volume (40 µL mg_S^{-1}) **Figure S5B**. The increase in electrolyte amount significantly extended the cycle life from approximately 50 (**Figure 3B,** main text) to 100 cycles. After the capacity declined sharply, the lithium anode was replaced and fresh electrolyte was added, resulting in a partial recovery of capacity that was sustained for more than 100 additional cycles. This observation provides direct evidence that the MC–S65 cathode structure and the cathode–electrolyte interphase (CEI) formed during the initial cycles remain stable and functional over prolonged cycling. Consequently, the overall cell lifetime is primarily constrained by anode and electrolyte consumption rather than cathode degradation.

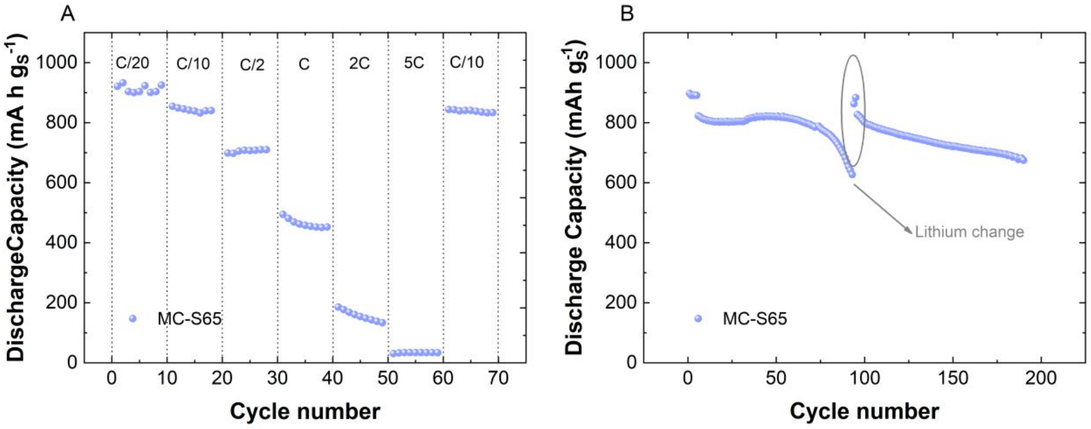

Figure S5. (A) Rate capability and (B) long-term cycling performance with lithium anode and electrolyte replacement of the MC-S65 cathode in a carbonate-based electrolyte.

Note 3: STACs using carbonate-based electrolyte

Barczak et al[1] presented a novel approach for sulfur infiltration into advanced carbon materials, termed sulfur-tuned advanced carbons (STACs). The synthesis process employed a steam-assisted sulfur insertion (SASI) method, which enables rapid and deep penetration of sulfur into the carbon's (pearl black (PB) 2000) porous structure while preserving its electrical conductivity. The STACs exhibited sulfur mass loadings of up to 85% and demonstrate tunable porosity by selectively filling different pore sizes. Sulfur is present, for the STACs with higher sulfur content, both in and out of the micropores. In their study, STACs were characterized using nitrogen adsorption, XPS, SEM-EDX, and TGA, confirming uniform sulfur distribution. The controlled infiltration process allows precise modulation of sulfur content and pore allocation, making STACs promising materials for energy storage and environmental applications. This method offers an environmentally friendly and scalable alternative to conventional sulfur infiltration techniques.

In this study, STAC materials were used to fabricate sulfur cathodes by combining the STAC composite with PVDF and C65 in an 8:1:1 weight ratio, which were then tested in carbonate-based electrolytes. **Figure S6** presents the first discharge process of the STAC cathodes, where a well-defined plateau appears between 2.25 V and 2.40 V, following the same trend observed for the MC-based materials specifically, the plateau capacity increases as the S/C ratio decreases. Given that a significant portion of sulfur remains outside the micropores in these cathodes (Barczak et al[1]), there is no clear indication that this external sulfur influences the observed electrochemical trends or mechanisms. Additionally, as shown in **Figure S7**, a control cathode composed of only sulfur and C65 does not exhibit the high potential discharge plateau, further suggesting that the process is surface-mediated. These findings strongly support the hypothesis that the first electrochemical process is not governed by sulfur outside the pore structure and that a microporous carbon framework is essential for this reaction to occur.

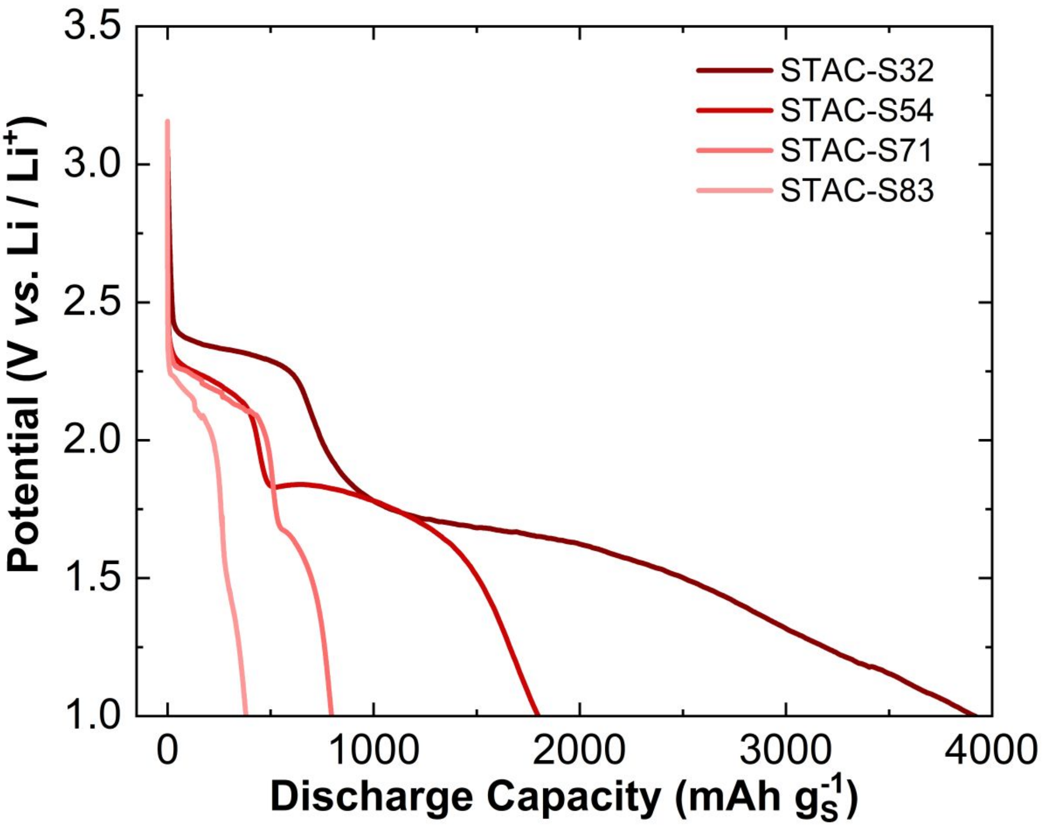

Figure S6. First discharge profile of the STACs material.

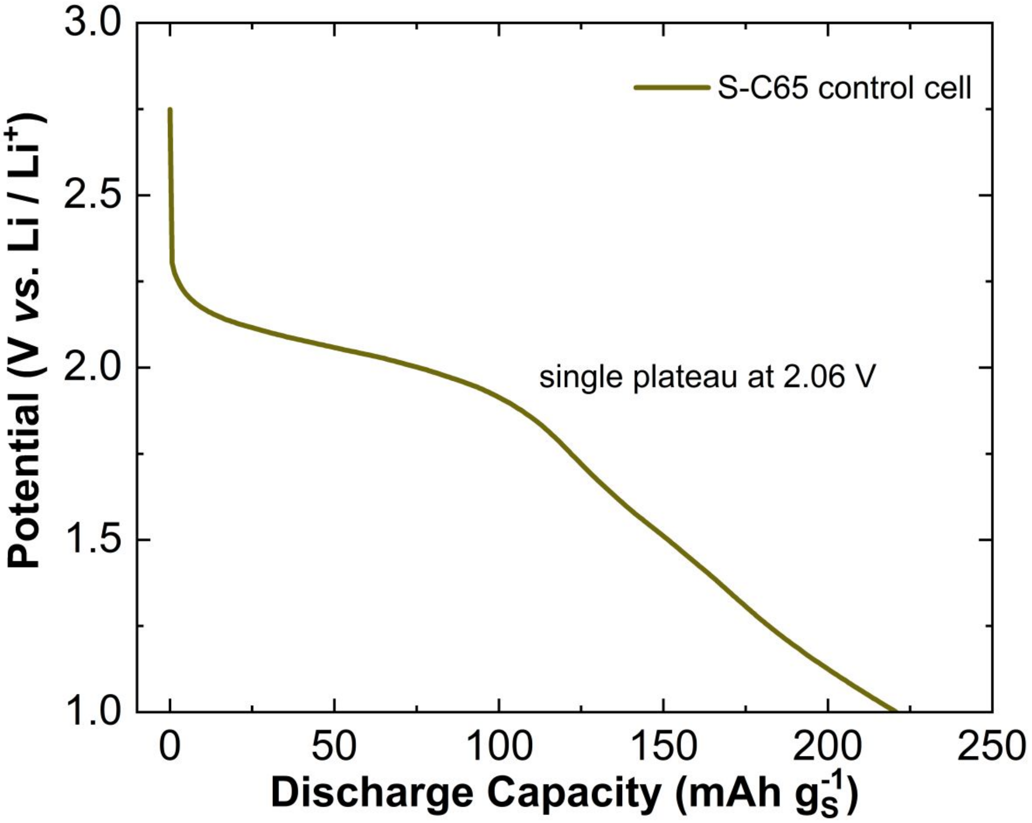

Figure S7. First discharge profile of the control cell.

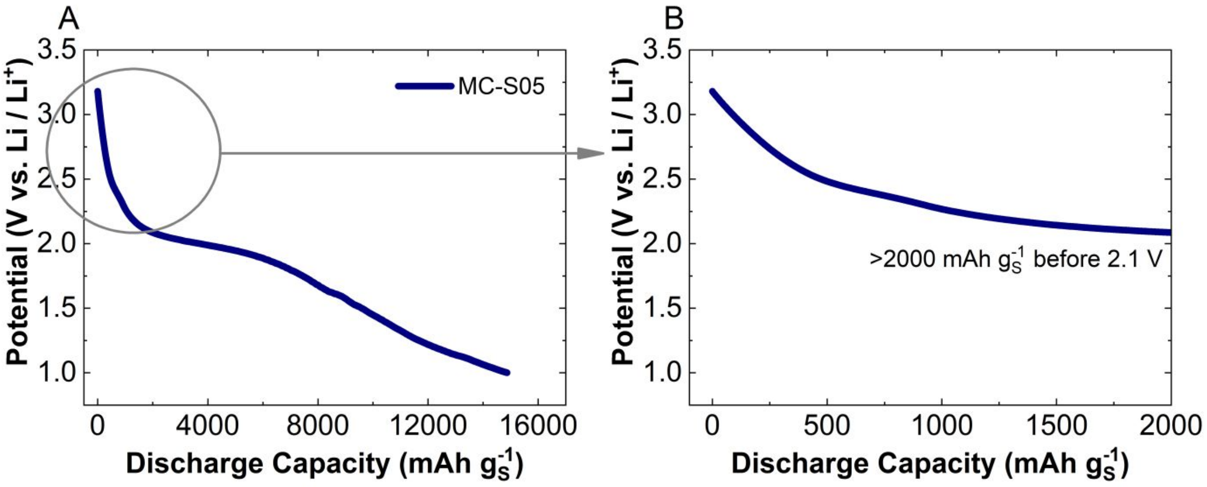

Figure S8. First discharge profile of the MC cathode with only 5 wt.% of sulfur. The first electrochemical process involves extreme capacities of 2000 mAh g^{-1} before the plateau at 2.0 V.

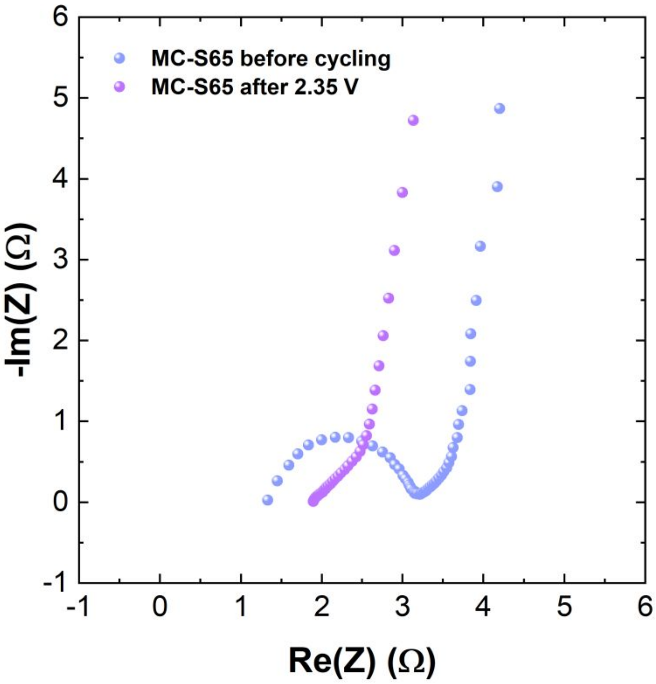

Figure S9: Nyquist spectra for the MC-S65 electrodes before and cycling and after the first plateau (2.1 V) in a symmetric configuration, cells measured at room temperature 30 °C.

Note 4: Kinetics considerations of CEI formation according to rate-dependent discharge

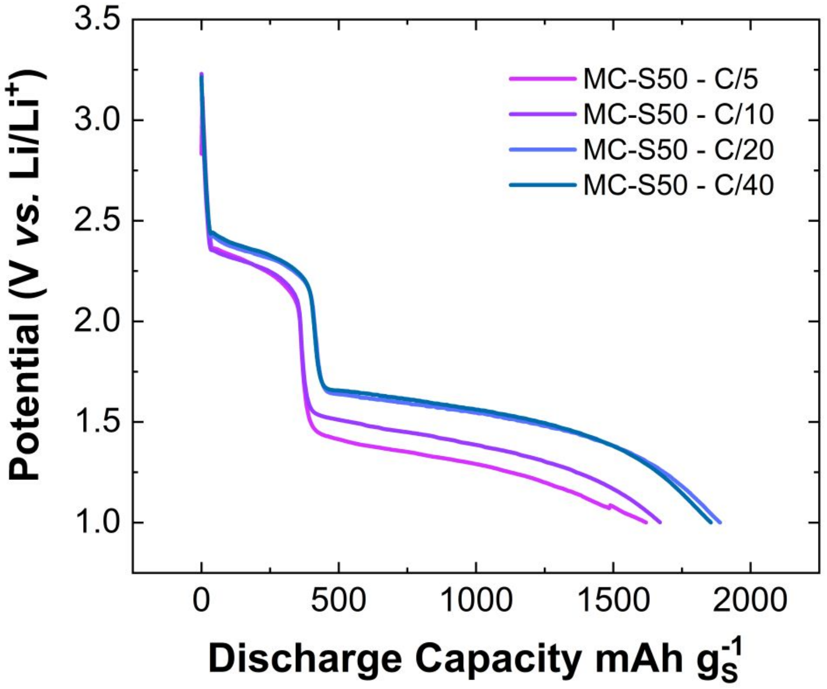

Figure S10: First discharge profile at different rates for the MC-S50 cathode.

The invariance of the first plateau's capacity across a wide range of C-rates, from C/5 to C/40 (**Figure S10**), provides critical insight into its kinetic nature. A process limited by the diffusion of a soluble species would exhibit a strong rate dependence; a longer plateau at slower rates would indicate more extensive polysulfide dissolution before CEI formation. The observed rate-independence, however, implies a self-limiting reaction governed by the fixed amounts where PS and solvent coexist within the micropores. Both reactions: PS formation and electrochemical nucleophilic attack must be a rapid, coupled process. This kinetic coupling ensures that CEI formation occurs efficiently, minimizing active material loss via dissolution and creating a consistent interface for the subsequent solid-state reduction.

Note 5: X-ray photoelectron (XPS) experiments

The core-level S 2*p* peak does not appear as a single peak but as a characteristic doublet. This is due to spin-orbit coupling, where the 2*p* orbital splits into two distinct energy levels: $2p_{3/2}$ and $2p_{1/2}$. The area ratio of these two peaks is fixed by their degeneracy at 2:1. Their separation, known as the spin-orbit splitting, is a constant value of around 1.18 eV for sulfur species. [2]

The high-resolution S 2*p* spectra were deconvoluted using a constrained fitting procedure based on established binding energies for sulfur species in Li-S battery electrodes.[2,3] The necessity for these four components was verified by attempting a fit with fewer contributions. A simplified two-component fit, for instance, was statistically inferior. It resulted in: 1) unphysically large full-width at half maximum (FWHM) values, and 2) a poor fit to the raw data, evidenced by a structured, non-random distribution of the residuals, indicating missing spectral features. The FWHM values for all components in the final four-peak fit were constrained to be below 1.3 eV, which is consistent with reported values for sulfur species in similar carbon-confined systems and confirms a chemically meaningful deconvolution.

The same fitting strategy was applied to all other spectra (C 1*s*, F 1*s*, and P 2*p*), where binding energies and FWHM values were carefully examined to ensure physically meaningful results.

Table S2. Type of function, binding energies (BE), full widths at half maximum (FWHM), and source of the C 1*s* deconvoluted spectra in **Figure 5** (main text) and **Figure S11-S14**.

**Species**	**Type**	**BE (eV)**	**FWHM (eV)**	**Source**
**C-C / C=C**	Voigt	284.6 ± 0.1	0.81 ± 0.05	C65, UMC
**C–O**	Voigt	285.1 ± 0.1	1.20 ± 0.05	UMC, electrolyte
**C–S**	Voigt	285.9 ± 0.1	1.20 ± 0.05	DMS
**CH_2**	Voigt	286.4 ± 0.1	1.23 ± 0.05	PVDF, CH_2-CHF (electrolyte decomposition)
**C–O–C/C–OH**	Voigt	284.3 ± 0.1	1.20 ± 0.05	Electrolyte
**C=O**	Voigt	288.6 ± 0.1	1.20 ± 0.05	Electrolyte
**O–C(=O)–O**	Voigt	290.4 ± 0.1	1.20 ± 0.05	DMC decomposition (Li_2CO_3)
**C–F**	Voigt	291.0 ± 0.1	1.15 ± 0.05	FEC, PVDF

Table S3. Type of function, binding energies (BE), full widths at half maximum (FWHM), and source of the F 1*s* deconvoluted spectra in **Figure 5** (main text) and **Figure S11-S14**.

**Species**	**Type**	**BE (eV)**	**FWHM (eV)**	**Source**
**Li–F**	Voigt	685.4 ± 0.2	1.5 ± 0.1	FEC decomposition
**C–F**	Voigt	686.4 ± 0.2	1.5 ± 0.1	PVDF, FEC
**POF**	Voigt	686.9± 0.2	1.5 ± 0.1	$LiPF_6$ decomposition
**CHF**	Voigt	688.0 ± 0.2	1.5 ± 0.1	CH_2-CHF (FEC decomposition)
**PF_6**	Voigt	688.7 ± 0.2	1.5 ± 0.1	$LiPF_6$

Table S4. Type of function, binding energies (BE), full widths at half maximum (FWHM), and source of the P 1*p* deconvoluted spectra in **Figure 4** (main text) and **Figure S8-S11**.

**Species**	**Type**	**BE (eV)**	**FWHM (eV)**	**Source**
**POF**	Voigt	135.5 ± 0.2	1.8 ± 0.1	$LiPF_6$ decomposition
**PF_6**	Voigt	136.9 ± 0.2	1.8 ± 0.1	$LiPF_6$

Table S5. Type of function, binding energies (BE), full widths at half maximum (FWHM), and source of the S 2*p* deconvoluted spectra in **Figure 5** (main text) and **Figure S11-S14**.

**Species**	**Type**	**BE (eV)**	**FWHM (eV)**	**Source**
**Li–S**	Voigt ($2p_{3/2}$)	161.5 ± 0.5	1.0 ± 0.1	Li_2S
**S–Li–S**	Voigt ($2p_{3/2}$)	163.2 ± 0.5	1.0 ± 0.1	Li_2S_x
**S–S**	Voigt ($2p_{3/2}$)	164.0 ± 0.5	1.0 ± 0.1	S_8 and Li_2S_x
**S–C**	Voigt ($2p_{3/2}$)	165.0 ± 0.5	1.0 ± 0.1	Electrolyte decomposition
**S–O**	Voigt ($2p_{3/2}$)	170.0 ± 0.5	1.0 ± 0.1	Oxidize sulfur from infiltration

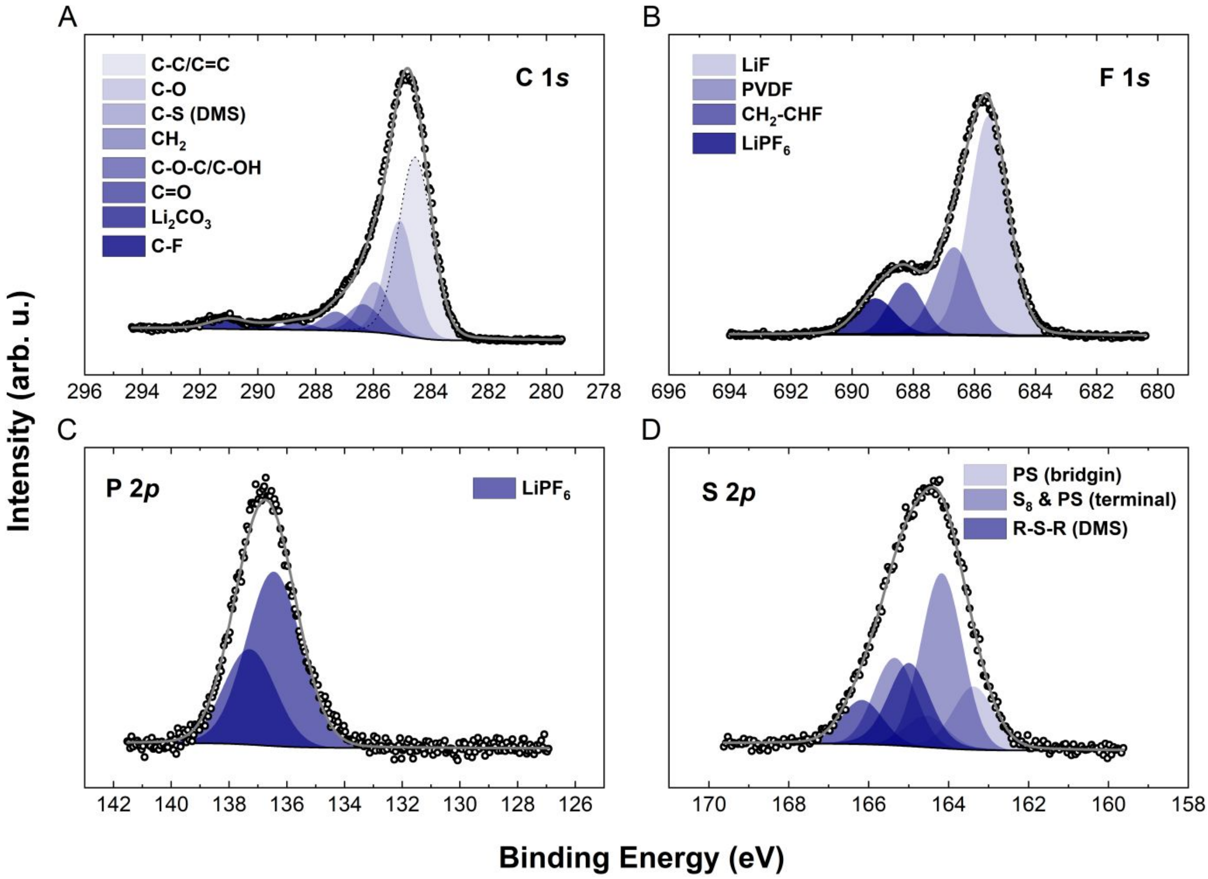

Figure S11. High-resolution X-ray photoelectron spectra and their deconvolution of the MC-S65 cathode after being discharged to 2.1 V; (A) C 1s. (B) F 1s, (C) P 2p, and (D) S 2p, where the black circles represent the acquired spectra, and the gray line shows the fit.

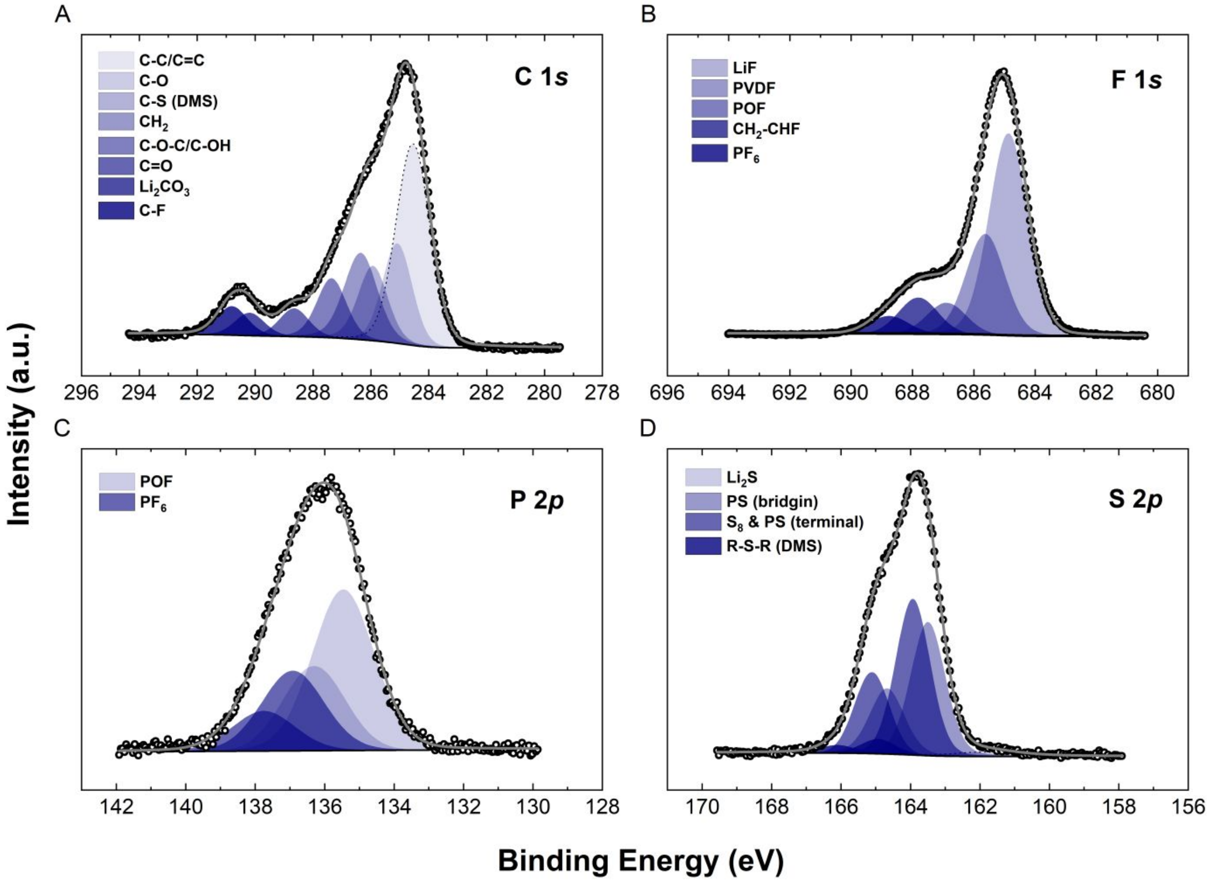

Figure S12. High-resolution X-ray photoelectron spectra and their deconvolution of the MC-S65 cathode after being discharged to 1.8 V; (A) C 1s. (B) F 1s, (C) P 2p, and (D) S 2p, where the black circles represent the acquired spectra, and the gray line shows the fit.

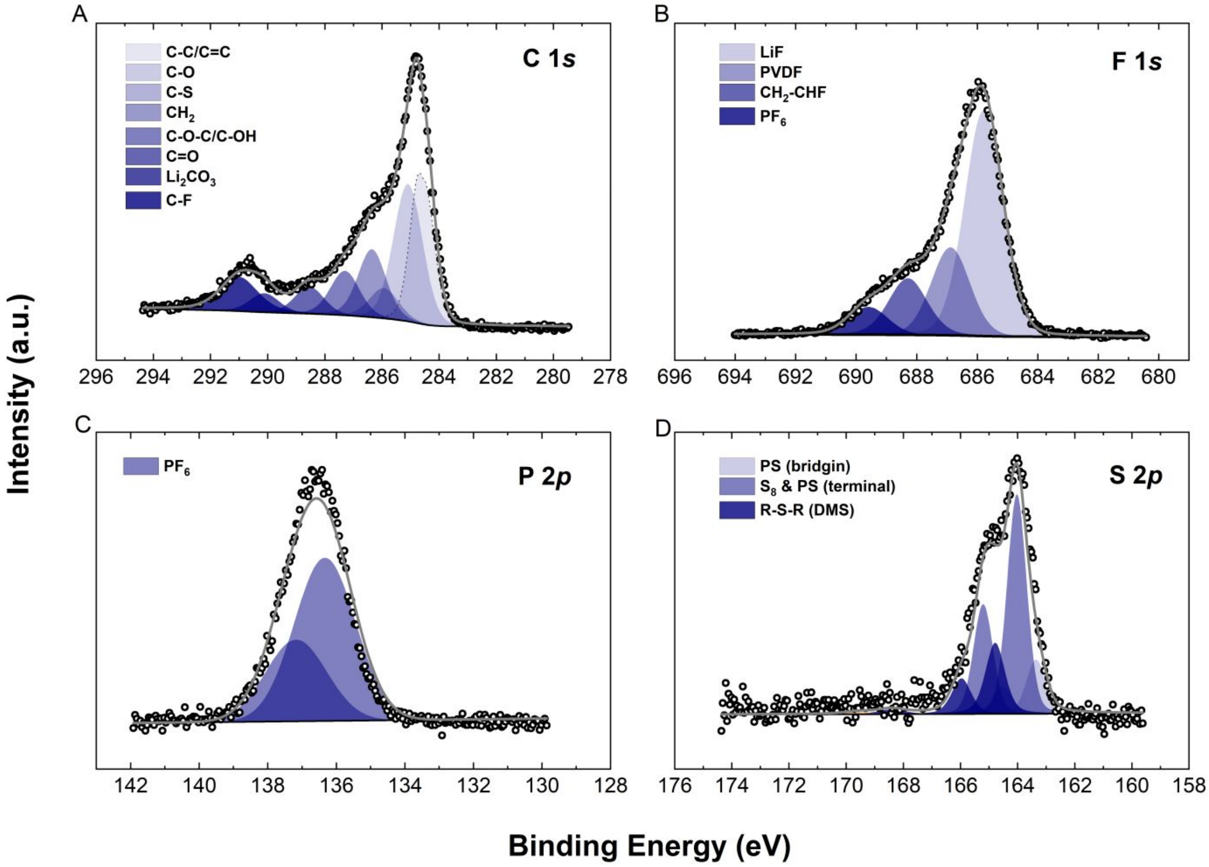

Figure S13 High-resolution X-ray photoelectron spectra and their deconvolution of the MC-S20 cathode after being discharged to 2.3 V; (A) C 1s. (B) F 1s, (C) P 2p, and (D) S 2p, where the black circles represent the acquired spectra, and the gray line shows the fit.

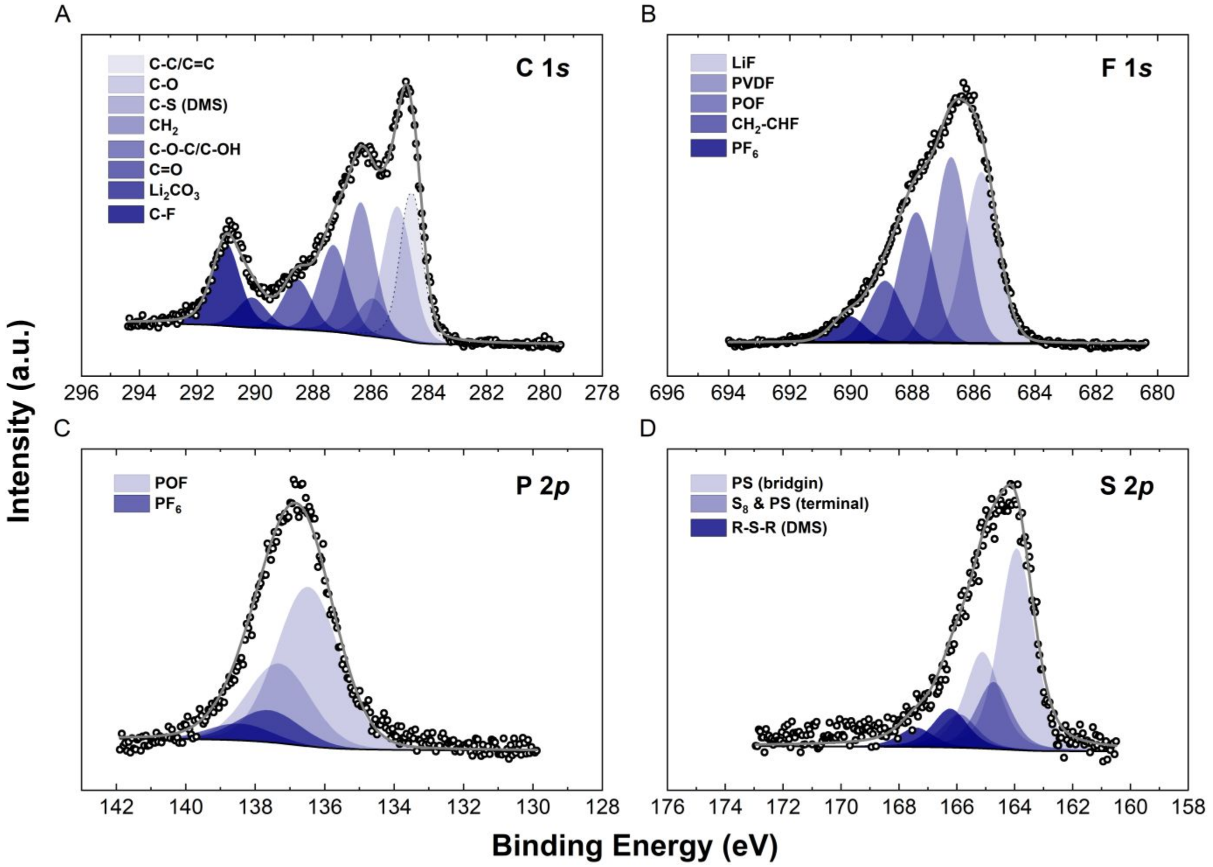

Figure S14. High-resolution X-ray photoelectron spectra and their deconvolution of the MC-S20 cathode after being discharged to 1.8 V; (A) C 1s. (B) F 1s, (C) P 2p, and (D) S 2p, where the black circles represent the acquired spectra, and the gray line shows the fit.

REFERENCES

[1] M. Barczak, M. Florent, S. S. Bhalekar, K. Kaneko, R. J. Messinger, T. J. Bandosz, *Adv Funct Mater* **2024**, *34*, DOI 10.1002/adfm.202310398.

[2] J. F. Moulder, J. Chastain, R. C. King, *Handbook of X-Ray Photoelectron Spectroscopy: A Reference Book of Standard Spectra for Identification and Interpretation of XPS Data*, Physical Electronics, **1995**.

[3] A. G. Shard, *Journal of Vacuum Science & Technology A* **2020**, *38*, DOI 10.1116/1.5141395.
